# Heterogeneous associations of socioeconomic status with metabolic disease in racial and ethnic subgroups in the United States: A cross-sectional cohort study in NHANES and *All Of Us*

**DOI:** 10.1371/journal.pone.0351075

**Published:** 2026-07-08

**Authors:** Sara J. Cromer, Julie E. Gervis, Sherri-Ann M. Burnett-Bowie, Chirag J. Patel

**Affiliations:** 1 Harvard Medical School, Boston, Massachusetts, United States of America; 2 Division of Endocrinology, Massachusetts General Hospital, Boston, Massachusetts, United States of America; 3 Center for Genomic Medicine; Massachusetts General Hospital, Boston, Massachusetts, United States of America; 4 Department of Biomedical Informatics, Harvard Medical School, Boston, Massachusetts, United States of America; Washington University in St Louis, IRAN, ISLAMIC REPUBLIC OF

## Abstract

**Background:**

Unfavorable socioeconomic status (SES) is associated with adverse health outcomes and is believed to at least partially mediate racial and ethnic health disparities such that some clinical risk algorithms now incorporate SES measures. However, whether the associations of improved SES with improved health are uniform across US racial and ethnic subpopulations is unknown.

**Methods and Findings:**

Among adult participants in the National Health and Nutrition Examination Survey 1999–2018 and the *All of Us* cohort v7, we used logistic regression to examine the association between SES measures (education and income) and type 2 diabetes (T2D) and obesity prevalence, comparing this association in the overall population and in subgroups of self-reported race and ethnicity. modeling SES in several ways to assess for race-by-SES interactions and non-linear or threshold effects.

**Results:**

Age-adjusted rates of T2D and obesity were highest among non-Hispanic Black, Mexican American, Other Hispanic, and Other/Multi-Racial participants and in those with lower SES. In stratified analyses, higher educational attainment and income were independently associated with lower rates of prevalent T2D and obesity among non-Hispanic White and Asian participants, with smaller or even reversed associations observed among other racial and ethnic groups, particularly non-Hispanic Black participants, in both datasets. Heterogeneity was confirmed by in race-by-socioeconomic interaction analyses. SES measures demonstrated variable patterns of association with disease (e.g., linear, threshold, or U-shaped associations) based on the SES measure, outcome, racial or ethnic group, and dataset used.

**Conclusions:**

The direction, magnitude, and shape of the association between SES and metabolic disease are heterogeneous across US racial and ethnic groups, SES measures selection and transformation, diseases, and datasets. To prevent biased estimates in both research and clinical calculators which increasingly attempt to incorporate SES, researchers and clinicians should examine for heterogeneity of associations between groups, particularly racial and ethnic groups.

## Introduction

Racial and ethnic minoritization and unfavorable socioeconomic status (SES) are associated with numerous adverse health outcomes in the United States (US), including metabolic diseases such as type 2 diabetes (T2D) and obesity [1–3]. With the growing movement to understand and study race as a social construct with health effects mediated by interpersonal, institutional, and structural racism resulting in inequitable treatment and distribution of resources, many scientists are working to replace race with more proximal and causal mediators of disparities such as SES in health research and even clinical care. However, our understanding of the role of SES, within and between groups in the US, is limited by several factors.

First, an underlying assumption in most studies relating SES with disease outcomes is that the association is homogeneous across population subgroups; stated differently, most analyses assume that an achievement of similar SES, for example attainment of a college degree, will confer a similar health benefit in all individuals. However, anecdotal evidence suggests reduced benefits of favorable SES among minoritized populations. For example, in a study of male graduates of Yale University in 1970, Black men had a mortality rate roughly triple that of their White peers by age 60 [4,5]. Similarly, a sociologist noted as early as the 1990s that while there seemed to be racial disparities in health across all socioeconomic strata, “for some indicators of health status…the racial gap becomes larger as SES increases” [6]. A large study demonstrated variable associations between SES and metabolic disease, namely obesity, between nations at differing stages of economic development [7], suggesting that the association of SES with disease is not monolithic and co-varies with other factors in the environment. However, this relationship has been less explored in contemporary subpopulations of the United States, although a growing body of work highlights “diminished returns” of favorable SES for cardiometabolic and other chronic diseases in marginalized populations [8–15], including groups with marginalization related to race, gender, sexual identity, and immigration status. Thus, the assumption that favorable SES benefits all population groups equally requires further study if we are to understand the intersection of SES and disease.

Second, there is no gold standard by which to measure and analyze SES and its association with disease. SES is a social construct which cannot be directly measured and is therefore frequently proxied by measures such as educational attainment, income, and employment status, either at the level of the individual (measuring personal SES) or over a geographic area (measuring the average SES of the individual’s neighborhood) [16–18]. The choice of which proxy measure or measures to use and how to transform them (e.g., continuous vs. categorized measures of income) varies substantially by study, resulting in limited reproducibility of results related to socioeconomic effects on disease [19,20]. It is hypothesized the means of transforming variables may lead to different conclusions ultimately and bias research findings, especially when these means are not standardized across studies [21,22].

In this report, we seek to inform both the intersectionality of race and ethnicity and SES and the impact of SES measure selection and transformation by leveraging ten cycles of the nationally representative National Health and Nutrition Examination Survey (NHANES) and the novel All of Us (AoU) cohort to examine the association of two objective and quantifiable proxy measures of SES – educational attainment and income– with T2D and obesity among individuals of different self-reported racial and ethnic identities. We hypothesized that individuals of different race and ethnicity, who are exposed to different social, cultural, environmental, and structural factors which may impact health, may experience different associations between SES and disease and that the association of SES with disease and the interaction of SES with race and ethnicity will differ based on both the SES measure and its means of transformation.

## Materials and methods

### Study population

We leveraged data from (1) the NHANES, a nationally representative sample of community-dwelling individuals in the US, from 1999−2018 (deidentified data accessed March-July 2023), and (2) the AoU cohort v8 (deidentified data accessed December 2023-April 2024 and April 2026, workspace identifier: aou-rw-781ef160), a national precision medicine initiative enrolling from the general US population, which has broad representation across racial and ethnic groups [23]. We excluded children (age < 18) and those with incomplete information regarding demographics (age, sex, race and ethnicity) or all elements used to define key exposures (education and income), outcomes (prevalent T2D and obesity, see below), or covariates (insurance status, smoking status).

In NHANES, we assigned all participants to a category based on self-reported race and ethnicity, including non-Hispanic White (NHW), non-Hispanic Black (NHB), Mexican American, Other Hispanic, non-Hispanic Asian (NHA), and Other/Multi-Racial race and ethnicity. Because NHA individuals were included among the Other/Multi-Racial category prior to 2011, we separated this category into pre- and post-2011 groups. In AoU, categories based on self-reported race and ethnicity were NHW, NHB, Hispanic, NHA, Multiracial, Other, and None of these.

### Exposure and outcomes definitions

The primary exposures were educational attainment and household income-to-poverty ratio (NHANES) or income (AoU). We chose these measures as they can be objectively measured and/or transformed to interpretable units (e.g., years of education, dollars of income) and are among the most commonly used proxy measures of SES [16,17]. In NHANES, we converted educational attainment from a categorical to a numeric variable, with each 1-unit increase representing approximately a 1–2 year increase in years of education, in order to preserve granularity, allow comparison over a period in which the survey categories were altered, and allow flexibility with modeling (S1 Table). Income-to-poverty ratio was provided within the NHANES and calculated based on annual household income and household size; it had a maximum value of 5.0, with all greater values set to 5.0. In AoU, we converted categorical educational attainment to numeric values matching those assigned in NHANES (S1 Table). In AoU, income was reported as a categorical variable and was transformed to a numeric variable (S2 Table) with each 1 unit increase representing a $25,000 increase in annual income, approximating the federal poverty level for a family of three in 2023 [24]. In both cohorts, a sensitivity analysis was performed categorizing education (no high school [NHANES only], less than high school, high school degree, some college [AoU only], or college degree).

The primary outcomes of this analysis were prevalent T2D and obesity. In NHANES, we defined T2D as the presence of diabetes (any of the following criteria: report of being diagnosed by a healthcare provider with diabetes, use of insulin or other medication for the participant-reported purpose of diabetes or blood sugar control, hemoglobin A1c (HbA1c) ≥ 6.5% (7.8 mmol/L), fasting blood sugar value ≥ 126 mg/dL (7.0 mmol/L), or random blood sugar value ≥ 200 mg/dL (11.1 mmol/L)) without evidence of type 1 diabetes (T1D; which was defined as diagnosis before age 30 years and treatment with insulin only). We defined obesity by the presence of any of the following: report of being told by a healthcare provider that the participant was overweight, use of a prescription medication for the participant-reported purpose of weight control, or body mass index (BMI) ≥ 30 kg/m^2^ among non-NHA participants or ≥ 27.5 kg/m2 among NHA participants [25].

In AoU, we defined T2D by either the presence of T2D based on self-report, the electronic health record (EHR) diagnoses-based “condition” dataset, or maximum HbA1c, fasting glucose, or random glucose consistent with a diagnosis of diabetes based on the criteria listed above; individuals with any evidence of T1D in the condition dataset were not considered to have T2D. We defined obesity based on either the presence of obesity in the condition dataset or a maximum recorded BMI, in either the AoU-collected data or EHR-based data, consistent with obesity based on the criteria listed above. Individuals missing all relevant data (from EHR records and anthropomorphic and blood measures) were excluded from the analysis.

### Associating SES with outcomes: statistical analysis

We conducted all NHANES analyses (including summary of baseline characteristics and all regression models) using weighting procedures to account for complex survey design, as recommended by the NHANES statistical guidance documents [26]. As the NHANES employs a multi-stage sampling approach, this including accounting for primary sampling units (PSU), sub-secondary sampling units, and individual-level exam weights to account for oversampling of sub-populations which varied by survey cycle [26,27]. For baseline characteristics, we reported mean and standard deviation and number and proportion for continuous and categorical measures, respectively. We calculated age-adjusted disease prevalence by standardizing, within each race and ethnicity category, to the projected U.S. population in 2000, consistent with National Center for Health Statistics practices in the NHANES [28].

We next performed multivariable logistic regression analyses examining the association between socioeconomic measures and disease outcomes. We performed all analyses initially in the entire sample, then in the entire sample with adjustment for race and ethnicity, and finally in subsamples restricted to single racial and ethnic groups. We adjusted all analyses for key covariates including age, sex, survey year (NHANES only), insurance type and stability (stably insured in the past year with private insurance, stably insured with other insurance, non-stably insured with private insurance (NHANES only), non-stably insured with other insurance (NHANES only), and uninsured; S3 Table), and smoking status (current, former, current or former unspecified (AoU), or never smoker). To test for statistical significance, we performed regression models including race and ethnicity-by-SES interactions to assess for heterogeneity of associations across groups, followed by modified, Satterthwaite-adjusted (NHANES, using survey procedures) and standard likelihood ratio tests examining whether the addition of interaction terms improved model fit.

We performed several sensitivity analyses, including logistic regression analyses using categorized, rather than continuously transformed, SES measures and using alternative model specifications (quasi-binomial, modified Poisson, and Poisson regressions) to confirm the associations between SES and disease outcomes in different racial and ethnic groups.

Following the evaluation for heterogeneity of the effect of SES on disease outcomes across racial and ethnic groups, we performed exploratory analysis examining whether one well-known cause of T2D, elevated BMI, mediated the association between SES and T2D to a similar degree in different groups, using structural equation models [29] to quantify the degree to which the association between SES measures and T2D was mediated through BMI.

We performed all analyses using R version 4.0.2 (R Core Team, 2020; Vienna, Austria), including the nhanesA, ggplot2, forestplot, fmsb, metafor, and lmtest packages [30–34].

### IRB approval and role of the funding source

The study leveraging de-identified, publicly available data from subjects who provided informed consent for inclusion in research repositories was deemed exempt from institutional review board (IRB) review by the Mass General Brigham IRB.

The funders of the study had no role in study design, data collection, data analysis, data interpretation, or writing of this report.

## Results

### Baseline characteristics

Between 1999 and 2018, 54,991 adults, representing a weighted population of approximately 217 million community-dwelling civilian adults, participated in the NHANES and had complete covariate data available (S1 Fig). Using weights to account for complex survey design, mean age was 41.2 years, 51.9% were female, and 67.9%, 11.2%, 8.3%, 5.6%, 2.4%, 3.1% and 1.5% identified as NHW, NHB, Mexican American, Other Hispanic, NHA, and Other/Multi-Racial race and ethnicity (pre- and post-2011), respectively (Table 1). In AoU, 404,990 individuals had complete covariate data available (S2 Fig). Mean age was 51.6 years, 64.0% were female, and 59.8%, 13.9%, 16.8%, 3.2%, 3.9%, 1.4%, and 1.0% identified as NHW, NHB, Hispanic, NHA, Multiracial, Other, and None of these races or ethnicities, respectively (Table 1). In both cohorts, NHW and NHA participants were more likely to report higher educational attainment and income-to-poverty ratio (S3 Fig). Educational attainment was modestly correlated with income (Pearson’s coefficient 0.45, p < 0.01 in NHANES; 0.45, p < 0.01 in AoU). Compared to survey-weighted NHANES, the AoU cohort was older and included more women, fewer individuals who identified as NHW and more who identified as NHB or Hispanic, and more individuals with the highest levels of educational attainment and income.

**Table 1:** Baseline characteristics of participants, including unweighted and weighted NHANES samples.

	NHANES 1999–2018: Unweighted (n = 54,991)	NHANES 1999–2018: Weighted (n = 216,871,589)		All of Us (n = 404,990)
Age, mean (SD)	49.43 (18.66)	41.77 (13.35)	Age, mean (SD)	51.58 (16.67)
Gender: Female, n (%)	28,527 (51.9)	112,606,704 (51.9)	Gender: Female, n (%)	257,010 (64.0)
NHANES Survey Year, n (%)				
1999-2000	4,687 (8.5)	18,026,031 (8.3)		–
2001-2002	5,240 (9.5)	20,716,338 (9.6)		–
2003-2004	4,934 (9.0)	20,199,151 (9.3)		–
2005-2006	4,931 (9.0)	20,913,734 (9.6)		–
2007-2008	5,884 (10.7)	21,324,803 (9.8)		–
2009-2010	6,162 (11.2)	21,770,096 (10)		–
2011-2012	5,501 (10.0)	22,272,803 (10.3)		–
2013-2014	6,070 (11.0)	23,609,064 (10.9)		–
2015-2016	5,865 (10.7)	23,679,578 (10.9)		–
2017-2018	5,717 (10.4)	24,359,993 (11.2)		–
Race/Ethnicity, n (%)			Race/Ethnicity, n (%)	
NHW	24,262 (44.1)	14,7481,527 (68)	NHW	240,203 (59.8)
NHB	11,474 (20.9)	24,348,427 (11.2)	NHB	55,750 (13.9)
Mexican American	4,503 (8.2)	12,136,002 (5.6)		–
Other Hispanic	3,004 (5.5)	5,200,063 (2.4)	Hispanic	67,364 (16.8)
NHA	9,564 (17.4)	17,819,748 (8.2)	NHA	12,918 (3.2)
Other/Multi-Racial including NHA (pre-2011)	1,331 (2.4)	6,657,433 (3.1)	Multiracial	15,628 (3.9)
Other/Multi-Racial (post-2011)	853 (1.6)	3,228,389 (1.5)	Other	5,654 (1.4)
			None of these	3,979 (1.0)
Educational Attainment (Continuous Educational Attainment Value), n (%)			Educational Attainment (Continuous Educational Attainment Value), n (%)	
Less Than 9th Grade (1)	6,047 (11.0)	12,828,321 (5.9)		–
Less Than High School Degree (2)	1,070 (1.9)	2,966,055 (1.4)	Less than a high school degree or equivalent (2)	27,580 (6.9)
9-11th Grade (3)	7,307 (13.3)	22,067,379 (10.2)		–
High School Degree or GED (4)	11,105 (20.2)	43,528,777 (20.1)	Highest Grade: Twelve Or GED (4)	65,070 (16.2)
High School Degree or GED or Some College or Associates Degree (5)	3,134 (5.7)	13,375,693 (6.2)		–
Some College or Associates Degree (6)	13,049 (23.7)	56,262,998 (25.9)	Highest Grade: College One to Three (6)	104,792 (26.1)
College Degree or Higher (7)	11,887 (21.6)	60,566,209 (27.9)	College graduate or advanced degree (7)	201,479 (50.2)
Do not know	83 (0.2)	157,130 (0.1)		–
Missing	1,295 (2.4)	5,091,022 (2.3)	PMI: Prefer Not To Answer	1,999 (0.5)
Refused	14 (0.0)	28,005 (0)	PMI: Skip	576 (0.1)
Continuous Educational Attainment,^a^ mean (SD)	4.70 (1.94)	5.36 (1.66)	Continuous Educational Attainment,^a^ mean (SD)	5.90 (1.50)
Household Income, n (%)			Household Income, n (%)	
	–	–	Annual Income: more 200k (9)	31,979 (8.0)
	–	–	Annual Income: 150k 200k (7.5)	23,528 (5.9)
$100,000 or more	5,476 (10.0)	32,324,052 (14.9)	Annual Income: 100k 150k (5.5)	49,275 (12.3)
$75,000-99,999	3,047 (5.5)	1,5606,953 (7.2)	Annual Income: 75k 100k (4)	38,726 (9.6)
$75,000 or more	3,714 (6.8)	20,321,616 (9.4)		–
$65,000-74,999	2,569 (4.7)	12,621,002 (5.8)	Annual Income: 50k 75k (3)	48,268 (12.0)
$55,000-64,999	3,184 (5.8)	14,437,844 (6.7)		–
$45,000-54,999	4,320 (7.9)	18,357,175 (8.5)		–
$35,000-44,999	5,210 (9.5)	20,068,404 (9.3)	Annual Income: 35k 50k (2)	34,373 (8.6)
$25,000-34,999	6,380 (11.6)	21,752,069 (10)	Annual Income: 25k 35k (2)	28,502 (7.1)
$20,000-24,999	4,146 (7.5)	12,934,326 (6)		–
Over $20,000	1,608 (2.9)	5,882,085 (2.7)		–
Under $20,000	443 (0.8)	1,054,752 (0.5)		–
$15,000-19,999	3,875 (7.0)	11,167,767 (5.1)	Annual Income: 10k 25k (1)	42,177 (10.5)
$10,000-14,999	3,988 (7.3)	10,684,859 (4.9)		–
$5,000-9,999	2,485 (4.5)	6,516,581 (3)	Annual Income: less 10k (1)	42,051 (10.5)
$0-4,999	1,157 (2.1)	3,193,678 (1.5)	PMI: Prefer Not To Answer	44,746 (11.1)
Missing	3,389 (6.2)	9,948,425 (4.6)	PMI: Skip	17,871 (4.5)
Income-to-Poverty Ratio, mean (SD)	2.52 (1.62)	3.28 (1.58)	Income (per $25,000), mean (SD)	3.67 (2.56)
Insurance Type and Stability,^b^ n (%)			Insurance Type and Stability,^b^ n (%)	
Stably Insured – Private	27,346 (49.7)	128,978,929 (59.5)	Stably Insured – Private	213,320 (53.1)
Stably Insured – Other	13,198 (24.0)	38,271,607 (17.6)	Stably Insured – Other	166,734 (41.5)
Unstably Insured – Private	1,798 (3.3)	7,732,641 (3.6)		–
Unstably Insured – Other	1,336 (2.4)	3,789,919 (1.7)		–
Uninsured	11,313 (20.6)	38,098,492 (17.6)	Uninsured	21,442 (5.3)

^a^See S1 Table

^b^See S2 TableNHANES = National Health and Nutrition Examination Survey; NHW = non-Hispanic White; NHB = non-Hispanic Black; NHA = non-Hispanic Asian.

### Association between SES and T2D and obesity prevalence

**Fig 1:**
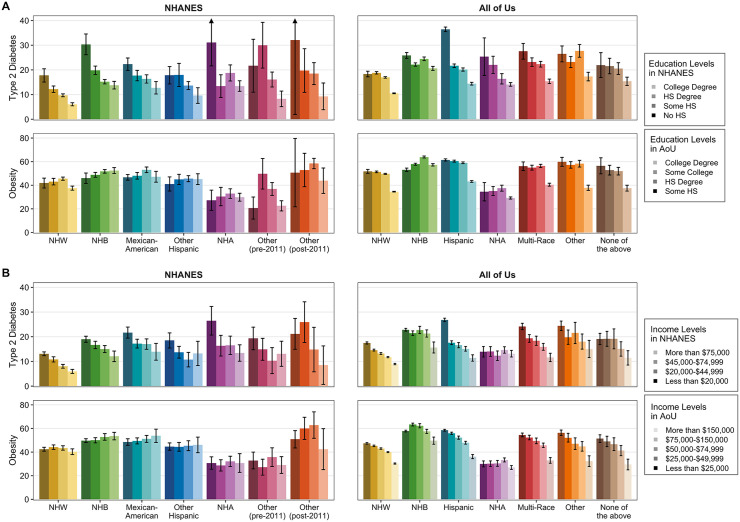
Age-standardized type 2 diabetes and obesity prevalence by race and ethnicity and by educational attainment (A) or income (B) in NHANES (left column) and AoU (right column).

In both cohorts, age-standardized prevalence of T2D was lower among NHW participants than among participants of other race and ethnicity (Fig 1). For example, NHB participants with a college degree had similar (NHANES) or even higher (AoU) T2D prevalence compared to NHW individuals with only some high school education (less than a high school degree). Similarly, age-standardized prevalence of obesity varied between racial and ethnic groups. NHB, Mexican American, and other Hispanic participants (NHANES) and NHB participants (AoU) with college degrees had higher rates of obesity than NHW individuals of any educational attainment, including those with less than a 9^th^ grade education.

**Fig 2:**
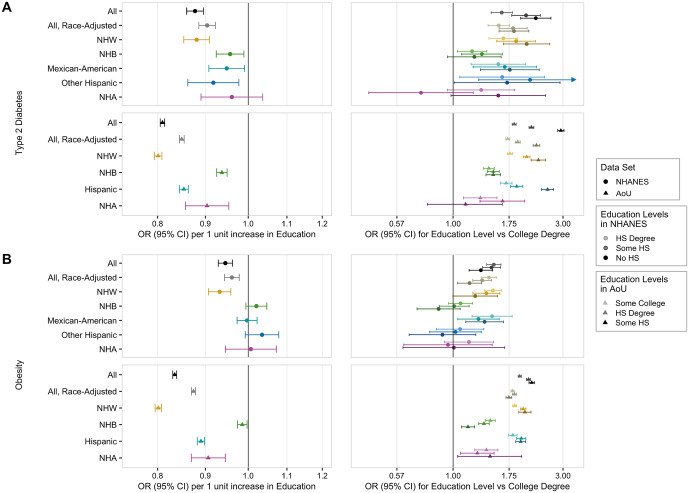
Education-Disease Associations in NHANES and AoU Populations. Forest plots of the adjusted association of educational attainment, modeled continuously (left column) or categorically (right column), with (A) type 2 diabetes and (B) obesity prevalence, in the population overall and in each racial and ethnic subgroup, in NHANES (circles) and AoU (triangles). For categorical analyses, the reference group is “College Degree or Higher” in both datasets.

In adjusted models, higher educational attainment was associated with lower prevalence of T2D in the entire population and in each race and ethnicity group except NHA (NHANES) or Other race or ethnicity (AoU; Fig 2A; S4 Table 4; S4A Fig). However, the effect size varied by race or ethnicity group. For instance, each 1 unit increase in continuous education measure (equivalent to approximately 1–2 years increase in educational attainment) was associated with a 12% decrease in T2D prevalence among NHW participants (OR 0.88 (95% CI 0.85, 0.91)) but a 4% decrease in prevalence among NHB participants (OR 0.96 (95% CI 0.92, 0.99)) in NHANES. In AoU, a similar discordance occurred with NHW participants experiencing a 19% decrease in T2D prevalence (OR 0.81 (95% CI 0.81, 0.81)) compared to only a 6% decrease in NHB participants (OR 0.94 (95% CI 0.93, 0.95)). Interaction analyses demonstrated significant race-by-SES interactions for NHB and Mexican-American individuals in NHANES and for all non-NHW racial and ethnic groups in AoU (Table 2). Modified (NHANES) and standard (AoU) likelihood ratio tests demonstrated that the addition of race-by-SES interactions significantly improved model fit in both cohorts (S5 Table).

**Table 2:** Race-by-SES Interactions: Interactions between race or ethnicity and socioeconomic status in fully adjusted regression models.

NHANES 1999–2018						All of Us			
	Educational Attainment		Income-to-poverty Ratio			Educational Attainment		Income	
	Diabetes	Obesity	Diabetes	Obesity		Diabetes	Obesity	Diabetes	Obesity
Race or ethnicity (Ref = NHW)					Race or ethnicity (Ref = NHW)				
NHB	1.55 (1.27, 1.88)	0.88 (0.73, 1.06)	1.72 (1.51, 1.97)	1.2 (1.08, 1.34)	NHB	0.56 (0.52, 0.61)	0.37 (0.34, 0.39)	1.60 (1.54, 1.67)	1.44 (1.39, 1.49)
Mexican-American	1.58 (1.28, 1.95)	0.93 (0.78, 1.10)	1.94 (1.61, 2.32)	1.24 (1.07, 1.44)		–	–	–	–
Other Hispanic	1.49 (1.04, 2.12)	0.71 (0.55, 0.91)	1.46 (1.11, 1.93)	1.06 (0.90, 1.26)	Hispanic	1.02 (0.95, 1.10)	0.76 (0.71, 0.81)	1.73 (1.66, 1.81)	1.53 (1.48, 1.58)
NHA	1.28 (0.86, 1.91)	0.32 (0.23, 0.47)	1.58 (1.18, 2.10)	0.41 (0.31, 0.53)	NHA	0.56 (0.40, 0.77)	0.27 (0.21, 0.35)	0.76 (0.67, 0.85)	0.44 (0.41, 0.48)
Other/Multi-Racial including NHA (pre-2011)	3.00 (1.84, 4.89)	0.93 (0.52, 1.66)	2.1 (1.40, 3.14)	0.72 (0.48, 1.07)	Multiracial	0.93 (0.78, 1.10)	0.77 (0.66, 0.89)	1.59 (1.48, 1.72)	1.30 (1.22, 1.38)
Other/Multi-Racial (post-2011)	1.95 (0.96, 3.95)	1.39 (0.73, 2.67)	1.59 (1.07, 2.35)	1.61 (1.17, 2.21)	Other	0.87 (0.71, 1.06)	0.83 (0.69, 0.99)	1.64 (1.47, 1.84)	1.41 (1.29, 1.55)
	–	–	–	–	None of these	0.91 (0.68, 1.23)	0.89 (0.69, 1.17)	1.35 (1.17, 1.56)	1.17 (1.04, 1.31)
Continuous SES (Education or Income)	0.89 (0.86, 0.92)	0.93 (0.91, 0.96)	0.88 (0.86, 0.91)	0.98 (0.95, 1.00)	Continuous SES (Education or Income)	0.78 (0.78, 0.79)	0.79 (0.78, 0.80)	0.87 (0.87, 0.88)	0.89 (0.89, 0.89)
Race-by-SES interactions					Race-by-SES interactions				
NHB*SES	**1.07 (1.03, 1.11)**	**1.10 (1.06, 1.14)**	**1.07 (1.03, 1.12)**	**1.08 (1.04, 1.11)**	NHB*SES	**1.24 (1.23, 1.26)**	**1.33 (1.32, 1.35)**	**1.05 (1.04, 1.07)**	**1.09 (1.08, 1.11)**
Mexican-American*SES	**1.05 (1.00, 1.11)**	**1.08 (1.04, 1.12)**	1.03 (0.97, 1.11)	1.04 (0.99, 1.10)		–	–	–	–
Other Hispanic*SES	1.02 (0.95, 1.10)	**1.09 (1.04, 1.14)**	1.06 (0.95, 1.18)	1.02 (0.96, 1.08)	Hispanic*SES	**1.08 (1.06, 1.09)**	**1.12 (1.10, 1.13)**	**0.97 (0.95, 0.98)**	**0.99 (0.98, 1.00)**
NHA*SES	1.07 (1.00, 1.14)	**1.08 (1.01, 1.15)**	1.02 (0.94, 1.12)	1.05 (0.98, 1.12)	NHA*SES	**1.14 (1.08, 1.20)**	**1.15 (1.11, 1.20)**	**1.11 (1.09, 1.14)**	**1.10 (1.08, 1.11)**
Other/Multi-Racial including NHA (pre-2011)*SES	0.93 (0.85, 1.03)	0.95 (0.86, 1.05)	0.98 (0.85, 1.12)	0.99 (0.88, 1.11)	Multiracial* SES	**1.10 (1.07, 1.13)**	**1.09 (1.07, 1.12)**	0.99 (0.97, 1.01)	1.00 (0.98, 1.01)
Other/Multi-Racial (post-2011)*SES	0.99 (0.86, 1.14)	0.99 (0.87, 1.11)	1.00 (0.83, 1.22)	0.92 (0.81, 1.05)	Other*SES	**1.13 (1.09, 1.17)**	**1.07 (1.04, 1.11)**	1.01 (0.97, 1.04)	0.98 (0.96, 1.01)
	–	–	–	–	None of these*SES	**1.07 (1.02, 1.13)**	1.04 (0.99, 1.08)	1.00 (0.96, 1.04)	0.98 (0.96, 1.01)

Models adjusted for age, sex, survey year (continuous, NHANES only), smoking status, and insurance status.

NHANES = National Health and Nutrition Examination Survey; NHW = non-Hispanic White; NHB = non-Hispanic Black; NHA = non-Hispanic Asian.

By contrast, the relationship between higher educational attainment and obesity was significant among NHW participants (OR 0.93 (95% CI 0.91, 0.96)) but not significant within any other racial or ethnic group in NHANES. The relationship between education and obesity in NHW participants in AoU was also significantly attenuated in almost all other groups in AoU (Fig 2B; S4 Table; S4B Fig). Interaction analyses demonstrated significant race-by-SES interactions for NHB, Mexican-American, Other Hispanic, and NHA individuals in NHANES and for NHB, Hispanic, NHA, Multiracial, and Other race individuals in AoU (Table 2). Likelihood ratio tests demonstrated that the addition of race-by-SES interactions significantly improved model fit in both cohorts (S5 Table).

**Fig 3:**
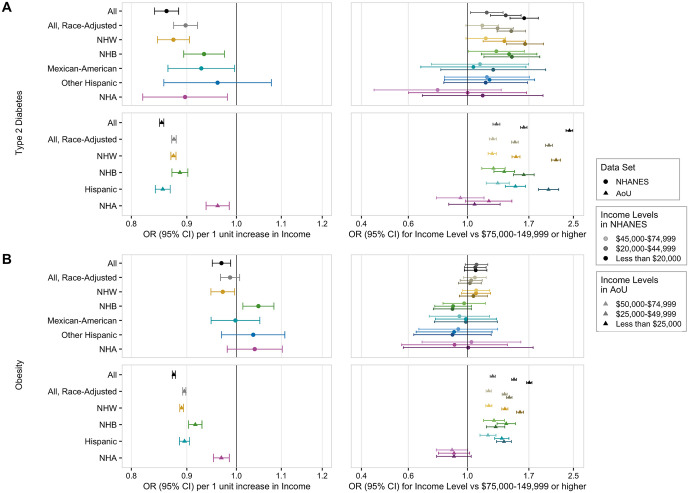
Income-Disease Associations in NHANES and AoU Populations. Forest plots of the adjusted association of income, modeled continuously (left column) or categorically (right column), with (A) type 2 diabetes and (B) obesity prevalence, in the population overall and in each racial and ethnic subgroup, in NHANES (circles) and AoU (triangles). For categorical analyses, the reference group is income ≥ $75,000 in NHANES and $75,000-149,999 in AoU. For interpretability, the highest income group in AoU (≥$150,000 per year), which did not exist in NHANES, is excluded from this Figure, but presented in S5 Fig.

Differences in the association between income and T2D prevalence in racial and ethnic subgroups were less pronounced with overlapping confidence intervals than observed between educational attainment and T2D, although the OR remained largest among NHW individuals in both datasets (Fig 3A; S4 Table; S5A Fig) with one exception: NHA participants in AoU experienced a smaller reduction in T2D prevalence compared to other participants (e.g., OR 0.96 (95% CI: 0.94, 0.98) for NHA v. OR 0.87 (95% CI: 0.87, 0.88) for NHW). Interaction analyses demonstrated significant race-by-SES interactions for NHB individuals in NHANES and for NHB, Hispanic, and NHA groups in AoU (Table 2). Likelihood ratio tests demonstrated that addition the of race-by-SES interactions significantly improved model fit in AoU but not in NHANES (S5 Table).

The racial and ethnic difference in effect size was perhaps most striking for the relationship between income and obesity in NHANES (Fig 3B; S4 Table; S5B Fig), with significant protective associations in the NHW cohort (OR 0.97 (95% CI 0.95, 0.996) not only attenuating but even reversing in the NHB cohort (OR 1.05 (95% CI 1.01, 1.08). We found no significant association for other racial and ethnic groups (Fig 2B) in NHANES. In AoU, this reversal of association was not observed; however, NHA participants had a significantly less protective association between income and obesity than other groups (OR 0.97 (95% CI: 0.95, 0.98) for NHA v. OR 0.89 (95% CI: 0.89, 0.89) for NHW; Fig 3B). Interaction analyses demonstrated significant race-by-SES interactions for NHB individuals in NHANES and for NHB, Hispanic, and NHA individuals in AoU (Table 2). Likelihood ratio tests demonstrated that the addition of race-by-SES interactions significantly improved model fit in both cohorts (S5 Table).

Sensitivity analyses examining categorized educational attainment and income demonstrated similar results, with greater protective effects of higher education and income among NHW individuals than among NHB individuals (Figs 2 and 3). However, categorical analyses demonstrated that modeling of either SES measure as a continuous value with a linear association with either outcome may not be appropriate across diverse racial and ethnic groups. For example, in AoU, decreasing categories of educational attainment were associated with a step-wise increase in risk for T2D in the overall cohort and in NHW individuals, but threshold effects existed in other racial and ethnic groups – anything less than a college degree was associated with approximately 50% increased odds of T2D among NHB individuals, with no differences between lesser levels of education, while having either a high school degree or at least some high school education resulted in approximately 80% increased odds of T2D for Hispanic individuals, compared to having a college degree, but having no high school education at all resulted in approximately 150% increased odds of T2D (Fig 2A). For other exposure-outcome pairs, their seemed to be U- or J-shaped curves, as well. For example, in NHANES, NHW individuals with a high school degree seemed to have higher risk for obesity compared to NHW individuals with either higher or lower educational attainment. Similarly, NHB individuals with a high school degree seemed to have higher risk for obesity than NHB individuals with either higher or lower educational attainment (Fig 2B).

Sensitivity analyses examining alternative modeling strategies (quasi-binomial, modified Poisson, and Poisson regressions) were consistent with the primary results (S6 and S7 Figs).

### Exploratory analysis: mediation of the association between SES and T2D by BMI

In addition to exploring heterogeneity of the association between SES and T2D across racial and ethnic groups, we aimed to explore whether this association was similarly mediated by differences in BMI between groups. We examined the proportion of the effect of socioeconomic measures on T2D prevalence which was mediated by differences in BMI, overall and within each population subgroup. We found the proportion of this association mediated through BMI varied by racial or ethnic subgroup. For example, 34.5% of the association between educational attainment and T2D prevalence was mediated through BMI among NHW participants in NHANES, while only 5–8% of this association was mediated through BMI among NHB, Mexican American, and Other Hispanic participants (Table 3). Similarly, 35.4% of the association between educational attainment and T2D prevalence was mediated through BMI among NHW participants in AoU, while only 4.3% and 12.0% of this association was mediated through BMI among NHB and Hispanic participants in AoU, respectively. While 22.6% of the association between income-to-poverty ratio and T2D prevalence was mediated through BMI among NHW participants in NHANES, only 0–16% of this association was mediated through BMI among NHB and Mexican American participants (Table 3).

**Table 3:** Mediation Analysis: Total effect, direct effect, and indirect effect (through body mass index) of educational attainment and income on type 2 diabetes prevalence and proportion mediated through body mass index, within the overall population and distinct racial and ethnic subpopulations.

	Educational Attainment				Income			
Race and Ethnicity Group	Total Effect	Direct Effect	Indirect Effect	Proportion Mediated	Total Effect	Direct Effect	Indirect Effect	Proportion Mediated
NHANES								
All	−0.146	−0.112	−0.034	**0.234**	−0.15	−0.123	−0.027	**0.18**
All-Race-adjusted	−0.112	−0.084	−0.028	**0.315**	−0.105	−0.085	−0.019	**0.266**
NHW	−0.137	−0.09	−0.047	**0.345**	−0.123	−0.096	−0.028	**0.226**
NHB	−0.044	−0.042	−0.002	**0.051**	−0.066	−0.066	0	**−0.001** ^**a**^
Mexican American	−0.069	−0.063	−0.005	**0.078**	−0.087	−0.081	−0.005	**0.061**
Other Hispanic	−0.101	−0.094	−0.006	**0.064**	−0.056	−0.047	−0.009	**0.159**
NHA	−0.037	−0.031	−0.007	**0.177**	−0.105	−0.113	0.008	**−0.074** ^**a**^
Other (pre-2011)	−0.174	−0.149	−0.025	**0.145**	−0.101	−0.091	−0.01	**0.096**
Other (post-2011)	−0.137	−0.118	−0.019	**0.139**	−0.095	−0.072	−0.023	**0.243**
AoU								
All	−0.227	−0.175	−0.052	**0.228**	−0.158	−0.113	−0.045	**0.284**
All-Race-adjusted	−0.175	−0.136	−0.039	**0.379**	−0.130	−0.093	−0.038	**0.486**
NHW	−0.242	−0.156	−0.086	**0.354**	−0.130	−0.085	−0.045	**0.348**
NHB	−0.063	−0.060	−0.003	**0.043**	−0.118	−0.090	−0.029	**0.242**
Hispanic	−0.164	−0.144	−0.020	**0.120**	−0.155	−0.130	−0.025	**0.164**
NHA	−0.096	−0.053	−0.043	**0.446**	−0.032	−0.026	−0.006	**0.199**
Multiracial	−0.155	−0.116	−0.039	**0.252**	−0.137	−0.095	−0.043	**0.310**
Other	−0.126	−0.081	−0.045	**0.358**	−0.120	−0.089	−0.031	**0.259**
None of these	−0.201	−0.158	−0.043	**0.216**	−0.158	−0.123	−0.035	**0.219**

^**a**^ Negative proportion mediated and proportion mediated >1 should be interpreted as the absence of mediation; both occur in the setting where direct and indirect effects (via the mediator, BMI) of the exposure (SES) on the outcome (type 2 diabetes) are in different directions.NHANES = National Health and Nutrition Survey; AoU = All of Us cohort; NHW = non-Hispanic White; NHB = non-Hispanic Black. NHA = non-Hispanic Asian; BMI = body mass index.

## Discussion

In this analysis examining the association between SES measures and metabolic disease in a nationally representative US population as well as a large national biobank, both overall and within distinct racial and ethnic groups, we found that the association between SES and disease varies in both magnitude and direction depending on the SES measure used (e.g., education or income), the means of transformation of the SES measure, the disease studied, and the population characteristics of the sample. Specifically, for example, we found that each 1–2 year increase in educational attainment was associated with as little as 4% and as much as 12–19% reduced odds of T2D, depending on racial or ethnic group and dataset. For each 1-unit increase in income-to-poverty ratio in NHANES, NHW individuals experienced 3% reduced odds of obesity while NHB individuals experienced 5% increased odds of obesity. Meanwhile, when SES measures were analyzed categorically, different patterns of association (linear, U-shaped, and threshold effects) were observed in different groups and for different diseases. Our findings suggest that monolithic analyses of the association between SES and disease within the US population may obscure variability which would be observed if examining associations within individual subpopulations, thus leading to an incomplete understanding of the populations at highest risk for disease as well as the potential impact of public health interventions.

A thorough understanding of the interplay of these two risk factors and the potential bias caused by analyzing socioeconomic measures homogeneously across populations is especially important as incorporation of SES in clinical prediction algorithms is already underway [35,36]. Further, some follow-up studies examining the inclusion of additional individual- and area-level SES measures in clinical algorithms have found statistically significant differences in the association of SES with disease, as well as differences in tool performance across racial and ethnic groups following the addition of these measures [37]. While efforts to move past “race-based medicine,” further incorporate the most proximal risk factors of disease, and capture sociocultural factors leading to disease are laudatory, we believe greater understanding of the role of SES is needed for its appropriate use in clinical care.

Most analyses examining socioeconomic health disparities examine diverse populations with a few models, reporting a single, non-stratified estimate across all groups. Although this may provide an overall, weighted average of the effect in the general population, our results suggest that it may obscure disparate associations which exist within subpopulations. As most US-based samples include a majority of NHW individuals, the overall result will be weighted to reflect predominantly the association observed within that population. As our analyses suggest that the association between SES and disease among NHW individuals is significantly different from that of other populations, this will systematically obscure important findings among minoritized groups. When reporting associations between SES and disease, future studies should examine for effect modification by race and ethnicity. Further research is also needed to examine whether the association of SES and disease is significantly modified by other factors, such as biological sex or gender, region, physical environment, or year [38,39]. SES may also impact diabetes risk differently in different population groups. In exploratory analyses notably limited in their interpretation by cross-sectional data collection, we observed that the proportion of the association of SES with T2D prevalence mediated through BMI varied substantially by race and ethnicity, highlighting differences in diabetes pathogenesis, with higher diabetes risk observed at lower BMI for non-White individuals, both in the US and across the world [40–42].

Limited studies have examined the intersection of race and ethnicity and SES on diabetes outcomes, but these have generally reported similar findings to our study, with SES having different associations with disease in different racial and ethnic groups. For example, a 2014 analysis using the National Health Information Survey examined the prevalence of T2D among poor and non-poor Black and White individuals living in poor and non-poor neighborhoods. The analysis found that a poor White person living in a poor neighborhood (highest risk group) had more than double the risk of diabetes compared to a non-poor White person living in a non-poor neighborhood (lowest risk group), while the analogous comparison among Black individuals (high individual and neighborhood risk v. low individual and neighborhood risk) was associated with only 17% increased risk of diabetes [43]. Similar to our findings, this suggests a different and greater impact of favorable SES among White individuals compared to Black individuals.

A larger body of work has examined the intersection of race and ethnicity and SES on obesity outcomes, demonstrating variable associations between SES and obesity rates in countries at different stages of the “obesity transition” [7]. For countries at earlier stages of this transition, generally lower-income and less industrialized countries, higher SES is associated with improved food security, more sedentary lifestyles, and higher rates of obesity [44]. However, countries may progress to later stages of the transition in which calorie-dense and sedentary environments affect the entire population, with those of higher SES being able to afford healthier foods and having the resources and time to devote to physical activity, thus leading to lower rates of obesity in high SES groups.

At the individual level, some authors in the field have articulated a theory of “ marginalization-related diminished returns” of improved SES among marginalized and immigrant populations [45]. For example, intersectionality of race or ethnicity and SES, has been reported for childhood BMI [10,11,46], asthma [47], heart disease [12], overall comorbidity or chronic disease burden [13–15,48] in a variety of cross-sectional and cohort studies with diminished benefits seen in marginalized groups which notably include racially and ethnically minoritized groups as in our study but also members of the LGBTQIA community and immigrants in both the USA and other nations [49]. For example, a study performed among individuals with osteoarthritis found that higher income was associated with reduced odds of being overweight or obese among White individuals but not among Black individuals [8], with a related study showing a similar interaction between educational attainment and sexual orientation on obesity, with LGBTQIA individuals experiencing reduced benefits from increasing SES compared to heterosexual individuals [9]. Similarly, several studies in the NHANES noted that the beneficial effects of educational attainment and employment on cardiometabolic health were attenuated in NHB adults compared to NHW adults [50]. and in men compared to women [51]. Related work in the AoU cohort also suggests that both SES effects and race-by-SES interactions may have disease-specific patterns [52]. These consistent findings provide a foundation through which the findings of our study exploring the intersectionality of SES and race or ethnicity on metabolic disease across diverse groups in large, nationally representative datasets may be interpreted. Importantly, they highlight that this phenomenon of marginalization-related diminished returns is not limited to race- or ethnicity-related marginalization and that it may be observed across diverse populations and diseases.

Our study supports prior evidence documenting marginalization-related diminished returns and suggests that different population groups within a single country may be at different stages of the “obesity transition.” We found that higher SES is associated with lower rates of obesity in NHW Americans but higher rates of obesity among NHB Americans, reflecting associations seen in countries at different stages of this phenomenon, due to different structural pressures influencing the environment, lifestyle, and disease risk of affected individuals. Within individual US states, heterogeneity in the association of individual- and area-level SES with BMI and mortality were also found in different racial and ethnic subgroups in the 1990s to early 2000s, further supporting that this same “obesity transition” phenomenon may exist within local populations [53–55]. Despite these earlier findings, only a minority of subsequent studies on the association of SES with metabolic disease examined the interaction between SES and other factors, with most studies continuing to analyze SES monolithically across populations [18,56,57].

Although the correlates of marginalization-related diminished returns likely vary by subpopulation, society, and disease state, several potential mechanisms have been identified. For example, while educational attainment is associated with decreased health-related social needs globally, studies have shown that this effect is diminished in Latino populations, where educational attainment is less protective against food insecurity, possibility related to differences in employment [58]. Similarly, cultural differences in dietary patterns or lifestyle behaviors, effects of structural racism such as segregation resulting in different physical environment exposures (e.g., adverse toxin or pollutant exposures), and chronic stress related to the experience of discrimination may continue to affect marginalized groups, particularly non-Hispanic Black individuals, despite attainment of greater SES [6,18,59–64].

While we consistently find heterogeneous associations of SES with disease across race and ethnicity groups, our findings revealed not only differences in magnitude and direction of SES effects on health but also differences in the pattern or shape of the association between these factors and disease in different data sets. This further illustrates the complexity of SES-disease modeling, whereby differences in dataset composition and selection bias, SES measure choice, and seemingly insignificant differences in variable transformation may lead to distinct patterns and magnitudes of association between SES and disease. For example, the magnitude and sometimes even direction of effect of the association with SES and disease differed in Asian populations in NHANES and AoU. We believe this is at least in part to selection bias in the AoU cohort, influencing both enrollment and data completeness in distinct patterns within race groups; for example, almost 80% of Asian AoU participants hold a college degree compared to approximately 50% in NHANES. As such, findings in NHANES, which allows for a nationally representative sample, may more closely describe true relationships within unselected Asian populations. Next, we see that different measures of SES – education and income – have different associations with disease outcomes, suggesting that the choice of socioeconomic measure should be carefully considered and interpreted, and if possible, standardized across research studies to improve interpretability and reproducibility of results. Finally, we see that differences in variable transformation meaningfully impact results. For example, in Fig 3 comparing the impact of income on disease across race and ethnicity groups, we observe differences (1) when SES is modeling continuously vs categorically, and (2) based on the means of categorizing income. Subtle differences in how income was transformed within datasets may contribute to these associations. For example, NHANES creates a continuous income-to-poverty ratio variable which has the advantage of incorporating household income, household size, and secular trends such as inflation to capture income in a more nuanced fashion. While we attempted to imitate this in AoU, transforming income to a continuous variable relative to the poverty limit in 2023, there are still subtle differences from NHANES. Most importantly, NHANES transforms all individuals with income-to-poverty ratio >5.0 as 5.0, creating an inability to capture variability within higher income groups. In categorical analyses, the income categories vary slightly between the two datasets, with an additional high-income category that is not available in NHANES. Lastly, while our measures were all absolute, other studies may model SES relatively within a population (e.g., increase in disease risk per SD change in an area-level SES measure); when modeling within subpopulations, this may lead to incomparable results between groups due to differences in underlying SES distribution. For all of these reasons, the use of more than one socioeconomic measure, when available, and carefully planned sensitivity analyses to examine the impact of variable transformation and modeling on study results may better capture diverse elements of SES, improve rigor and reproducibility of SES-related analysis, and have greater predictive value for health outcomes, as seen in previous studies [65,66].

Finally, it is important to highlight that racial and ethnic disparities in T2D and obesity prevalence far outweigh socioeconomic disparities in our study, with NHB individuals with college degrees having a similar or greater age-standardized prevalence of T2D as NHW individuals with less than a high school degree. Previous studies have similarly documented that SES only partially mediates the association between race and ethnicity and health outcomes [67,68]. Although efforts to improve socioeconomic barriers to care are critical, they are not sufficient to overcome racial and ethnic disparities in the United States which arise from manifold factors in the environment – including direct and indirect effects of structural racism affecting individuals’ physical environments, chronic stress, and access to healthcare and required elements of a healthy lifestyle – that are not captured by simple socioeconomic adjustment.

This study has numerous strengths including large sample size in a nationally representative population. However, our findings must be interpreted considering the study design and data sources. NHANES is a cross-sectional survey, and direct and mediated associations of prevalent factors measured at a single time point may not be causal; however, the age at which participants attain their SES, especially educational attainment, is most likely to come before T2D onset and study entry (particularly as our population included only adults, and T2D cases must have had diabetes onset after age 30), making reverse causality less likely. The categories of race and ethnicity used in NHANES changed in 2011−2012 such that NHA individuals were included with the “Other/Multi-Racial” category before that time but not subsequently; this may lead to diverse populations included within the pre-2011 Other/Multi-Racial category and decreased sensitivity of obesity designations, as the NHA-specific cutoff was applied only to those self-reporting as NHA, a category which only existed beginning in 2011−2012. The conversion of educational attainment from a categorical to a numeric variable assumes a linear relationship between educational attainment and health outcomes and approximately equal distance or effect between adjacent categories of educational attainment; we performed sensitivity analyses using categorical education and income to confirm our results. In NHANES, the income-to-poverty measures have a maximum value of 5.0, treating all participants with the highest income levels as equal; however, this measure has the strength of being relatively comparable over time, incorporating broad economic changes and inflation, which is not true for categorical income Our NHANES sample terminated in 2018 due to irregularities in field operations during the 2019−2020 cycle related to the COVID-19 pandemic; although the sample has been combined with the previous sample and re-weighted to create an overall nationally representative sample, there are concerns that this sample may not be representative within population subgroups, including racial and ethnic subgroups relevant to this work [27,69]. Mediation analyses rely on multiple strong assumptions, particularly that all potential confounding between the exposure, mediator, and outcome has been accounted for, and we cannot be sure that these assumptions are met in this analysis; results of the mediation analysis should be interpreted cautiously. Although the findings in NHANES and AoU were overall consistent, showing heterogeneity in the association between SES and metabolic disease, with NHW individuals generally receiving the greatest relative benefit from increased SES, differences observed between the two analyses may be due to a number of factors including temporal differences in recruitment of participants, minor differences in collection and transformation of the key SES exposure variables, and selection bias in AoU. For example, the NHA cohort in AoU had very high levels of educational attainment and income, suggesting a selected population which is not representative of the US population and demonstrated a different pattern of association between SES and metabolic disease, albeit with overlapping confidence intervals. However, the consistent directionality of effects, which remain distinct from the NHW population in both datasets, suggests that our primary findings of heterogeneous effects of SES across racial and ethnic groups are robust even in the face of significant selection bias and that these patterns persist even in high-SES groups. Lastly, our analyses are not adjusted for multiple testing.

In conclusion, we found that the association between measures of SES and T2D and obesity outcomes vary in both magnitude and direction between racial and ethnic subgroups in the United States over the past two decades and currently. Similarly, choice of SES measure and transformation and modeling strategies revealed small but meaningful differences in described associations. In studies examining socioeconomic effects on disease outcomes, associations between SES and clinical outcomes should not be analyzed monolithically nor assumed to be consistent across subpopulations living in different cultural contexts. We recommend stratified analyses, with robust sensitivity analyses examining the effects of SES modeling choices on outcomes, to document heterogeneity between groups and improve the reproducibility of SES-related health research.

## Supporting information

S1 Table Conversion of categorical educational attainment to a continuous variable. (DOCX)

S2 Table Conversion of categorical income to a continuous variable (AoU only). (DOCX)

S3 Table Insurance type and stability variable classification. (DOCX)

S4 Table Adjusted association of educational attainment and income with type 2 diabetes and obesity prevalence. (DOCX)

S5 Table Model diagnostics. (DOCX)

S1 Fig Flow diagram of participant exclusion from the NHANES analysis. (PDF)

S2 Fig Flow diagram of participant exclusion from the All of Us cohort analysis. (PDF)

S3 Fig Distribution of educational attainment and income by race or ethnicity. (PNG)

S4 Fig Expansion of Figure 2, including underrepresented racial and ethnic groups. (TIFF)

S5 Fig Expansion of Figure 3, including underrepresented racial and ethnic groups. (TIFF)

S6 Fig Sensitivity analyses using alternate modeling assumptions for continuous education associations. (TIFF)

S7 Fig Sensitivity analysis using alternate modeling assumptions for continuous income measure associations. (TIFF)
