# Supplementary material for: Heterogeneous associations of socioeconomic status with metabolic disease in racial and ethnic subgroups in the United States: A cross-sectional cohort study in NHANES and All Of Us
Source: PLoS One. 2026 Jul 8;21(7):e0351075. doi: 10.1371/journal.pone.0351075 (PMC13345235; doi:10.1371/journal.pone.0351075)
Supplement: S1 Table — (DOCX) [file pone.0351075.s001.docx]

**S1 Table: Conversion of categorical educational attainment to a continuous variable.**

|  |  | NHANES |  |  | AoU |  |
| --- | --- | --- | --- | --- | --- | --- |
| Continuous Educational Attainment Value | Categorical Educational Attainment Level | NHANES Educational Attainment Category | NHANES Survey Years Used | Approximate Years of Education | AoU Educational Attainment Category | Approximate Years of Education |
| 1 | No High School | Less Than 9th Grade | 1999-2016 | 0-8 |  |  |
| 2 | Less than High School | Less Than High School Degree | 2017-2018 | 0-11 | Less than High School | 0-11 |
| 3 | Less than High School | 9-11th Grade | 1999-2016 | 9-11 |  |  |
| 4 | High School Degree | High School Degree or GED | 1999-2016 | 12 | High School Degree | 12 |
| 5 | High School Degree | High School Degree or GED or Some College or Associate Degree | 2017-2018 | 12-15 |  |  |
| 6 | High School Degree | Some College or Associate Degree | 1999-2016 | 13-15 | Some College | 13-15 |
| 7 | College Degree or Higher | College Degree or Higher | 1999-2018 | 16+ | College Degree or Higher | 16+ |
