# Supplementary material for: Heterogeneous associations of socioeconomic status with metabolic disease in racial and ethnic subgroups in the United States: A cross-sectional cohort study in NHANES and All Of Us
Source: PLoS One. 2026 Jul 8;21(7):e0351075. doi: 10.1371/journal.pone.0351075 (PMC13345235; doi:10.1371/journal.pone.0351075)
Supplement: S2 Table — (DOCX) [file pone.0351075.s002.docx]

**S2 Table: Conversion of categorical income to a continuous variable (AoU only).**

| Continuous Income Value | AoU Income Categories | Multiples of $25,000 (FPL for family of 3 in 2023) |
| --- | --- | --- |
| 1 | $0-9,999  $10,000-24,999 | 0-1 |
| 2 | $25,000-34,999  $35,000-49,999 | 1-2 |
| 3 | $50,000-74,999 | 2-3 |
| 4 | $75,000-99,999 | 3-4 |
| 5.5 | $100,000-149,999 | 4-6 |
| 7.5 | $150,000-199,999 | 6-8 |
| 9 | $200,000 or more | 8+ |
