# Supplementary material for: Heterogeneous associations of socioeconomic status with metabolic disease in racial and ethnic subgroups in the United States: A cross-sectional cohort study in NHANES and All Of Us
Source: PLoS One. 2026 Jul 8;21(7):e0351075. doi: 10.1371/journal.pone.0351075 (PMC13345235; doi:10.1371/journal.pone.0351075)
Supplement: S3 Table — (DOCX) [file pone.0351075.s003.docx]

**S3 Table: Insurance type and stability variable classification.**

| Insured Currently | Insurance Type | Uninsured in Past Year (NHANES only) | NHANES Insurance Type and Stability Variable Designation | AoU Insurance Type and Stability Variable Designation |
| --- | --- | --- | --- | --- |
| Yes | Private | Yes | Stably Insured-Private | Stably Insured-Private |
| Yes | Other^a^ | Yes | Stably Insured-Other | Stably Insured-Other |
| Yes | Private | No | Unstably Insured-Private | - |
| Yes | Other^a^ | No | Unstably Insured-Other | - |
| No | - | - | Uninsured | Uninsured |

^a^ Other insurance types include: Medicare, Medi-Gap, Medicaid, SCHIP, military, Indian Health Service, state-sponsored, other government-sponsored, and single service plans. If a participant reported both private and other types of insurance, they were coded as privately insured.
