# Supplementary material for: Heterogeneous associations of socioeconomic status with metabolic disease in racial and ethnic subgroups in the United States: A cross-sectional cohort study in NHANES and All Of Us
Source: PLoS One. 2026 Jul 8;21(7):e0351075. doi: 10.1371/journal.pone.0351075 (PMC13345235; doi:10.1371/journal.pone.0351075)
Supplement: S4 Table — (DOCX) [file pone.0351075.s004.docx]

**S4 Table: Adjusted association of educational attainment and income with type 2 diabetes and obesity prevalence**, overall and in strata of race and ethnicity (depicted in Figures 2 and 3), per 1 unit increase in continuous educational achievement of income (see Supplemental Tables 1-2).

|  | SES Measure: Educational Attainment | SES Measure: Educational Attainment | SES Measure: Income-to-Poverty Ratio or Income | SES Measure: Income-to-Poverty Ratio or Income |
| --- | --- | --- | --- | --- |
| **NHANES** | Outcome: T2D | Outcome: Obesity | Outcome: T2D | Outcome: Obesity |
| All | 0.88 (0.86, 0.90) | 0.95 (0.93, 0.96) | 0.86 (0.84, 0.88) | 0.97 (0.95, 0.99) |
| All-Race-adjusted | 0.90 (0.89, 0.92) | 0.96 (0.94, 0.98) | 0.90 (0.88, 0.92) | 0.99 (0.97, 1.01) |
| NHW | 0.88 (0.85, 0.91) | 0.93 (0.91, 0.96) | 0.87 (0.85, 0.90) | 0.97 (0.95, 1.00) |
| NHB | 0.96 (0.92, 0.99) | 1.02 (0.99, 1.05) | 0.93 (0.89, 0.97) | 1.05 (1.01, 1.08) |
| Mexican American | 0.95 (0.91, 0.99) | 1.00 (0.97, 1.02) | 0.93 (0.86, 1.00) | 1.00 (0.95, 1.05) |
| Other Hispanic | 0.92 (0.86, 0.98) | 1.03 (0.99, 1.08) | 0.96 (0.86, 1.08) | 1.04 (0.97, 1.11) |
| NHA | 0.96 (0.89, 1.04) | 1.01 (0.95, 1.07) | 0.90 (0.82, 0.98) | 1.04 (0.98, 1.10) |
| Other (pre-2011) | 0.85 (0.77, 0.95) | 0.93 (0.85, 1.02) | 0.92 (0.78, 1.09) | 1.02 (0.91, 1.15) |
| Other (post-2011) | 0.89 (0.78, 1.02) | 0.95 (0.84, 1.08) | 0.92 (0.77, 1.11) | 0.94 (0.82, 1.09) |
| **AoU** |  |  |  |  |
| All | 0.81 (0.81, 0.81) | 0.83 (0.83, 0.84) | 0.85 (0.85, 0.86) | 0.88 (0.87, 0.88) |
| All-Race-adjusted | 0.85 (0.84, 0.85) | 0.87 (0.87, 0.88) | 0.88 (0.87, 0.88) | 0.90 (0.89, 0.90) |
| NHW | 0.80 (0.79, 0.81) | 0.80 (0.80, 0.81) | 0.87 (0.87, 0.88) | 0.89 (0.89, 0.89) |
| NHB | 0.94 (0.93, 0.95) | 0.99 (0.97, 1.00) | 0.89 (0.87, 0.90) | 0.92 (0.90, 0.93) |
| Hispanic | 0.85 (0.84, 0.86) | 0.89 (0.88, 0.90) | 0.86 (0.84, 0.87) | 0.90 (0.89, 0.91) |
| NHA | 0.90 (0.86, 0.95) | 0.91 (0.87, 0.95) | 0.96 (0.94, 0.98) | 0.97 (0.95, 0.98) |
| Multiracial | 0.86 (0.84, 0.89) | 0.88 (0.86, 0.90) | 0.87 (0.85, 0.89) | 0.90 (0.88, 0.92) |
| Other | 0.89 (0.85, 0.92) | 0.86 (0.83, 0.89) | 0.88 (0.85, 0.92) | 0.89 (0.86, 0.92) |
| None of these | 0.83 (0.79, 0.88) | 0.83 (0.79, 0.87) | 0.86 (0.82, 0.90) | 0.88 (0.85, 0.92) |
