# Supplementary material for: Heterogeneous associations of socioeconomic status with metabolic disease in racial and ethnic subgroups in the United States: A cross-sectional cohort study in NHANES and All Of Us
Source: PLoS One. 2026 Jul 8;21(7):e0351075. doi: 10.1371/journal.pone.0351075 (PMC13345235; doi:10.1371/journal.pone.0351075)
Supplement: S5 Table — (DOCX) [file pone.0351075.s005.docx]

**S5 Table: Model diagnostics:** P values from modified, Satterthwaite-adjusted likelihood ratio tests (NHANES) and likelihood ratio tests (AoU) testing whether addition of race-by-SES interactions to fully adjusted models improve model fit.

| Model outcome | Model SES exposure | NHANES | AoU |
| --- | --- | --- | --- |
| T2D | Educational Attainment | p = 0.023 | p <0.001 |
| T2D | Income | p = 0.314 | p <0.001 |
| Obesity | Educational Attainment | p <0.001 | p <0.001 |
| Obesity | Income | p = 0.024 | p <0.001 |
