# Supplementary figures and images for: Heterogeneous associations of socioeconomic status with metabolic disease in racial and ethnic subgroups in the United States: A cross-sectional cohort study in NHANES and All Of Us
Source: PLoS One. 2026 Jul 8;21(7):e0351075. doi: 10.1371/journal.pone.0351075 (PMC13345235; doi:10.1371/journal.pone.0351075)

**S1 Fig: Flow diagram of participant exclusion from the NHANES analysis.**

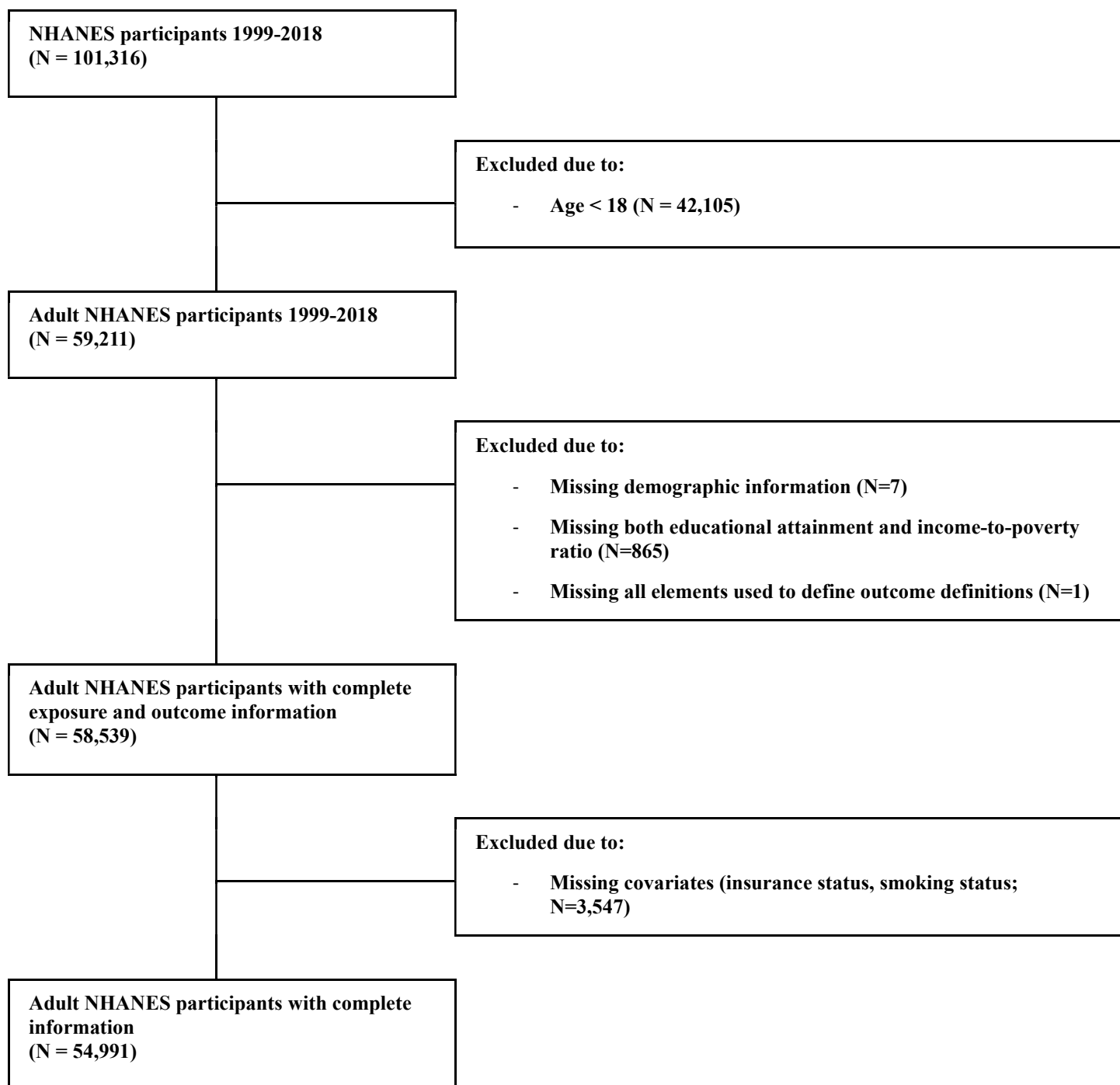

Supplement: S1 Fig — (PDF) [file pone.0351075.s006.pdf]

**S2 Fig: Flow diagram of participant exclusion from the All of Us cohort analysis.**

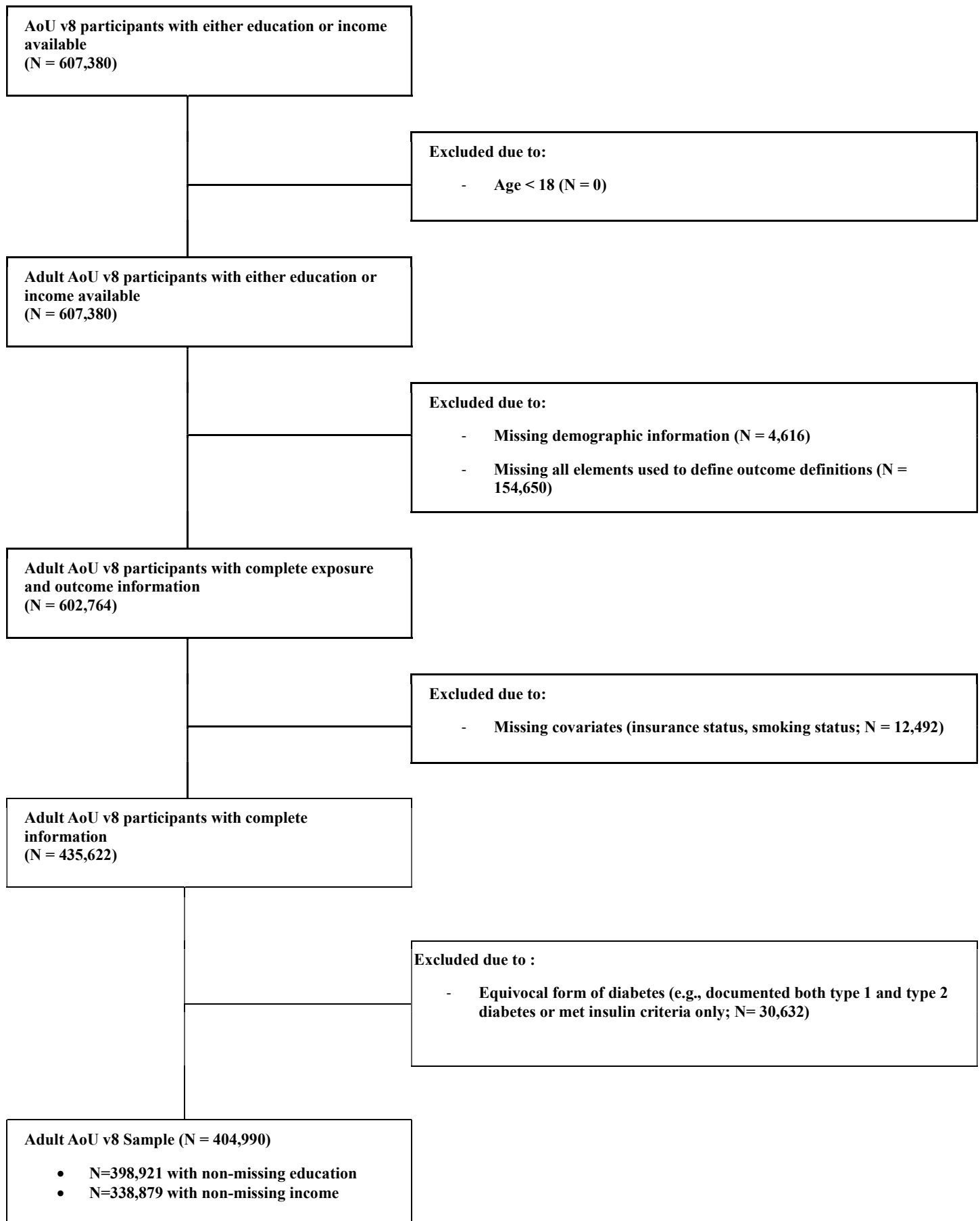

Supplement: S2 Fig — (PDF) [file pone.0351075.s007.pdf]

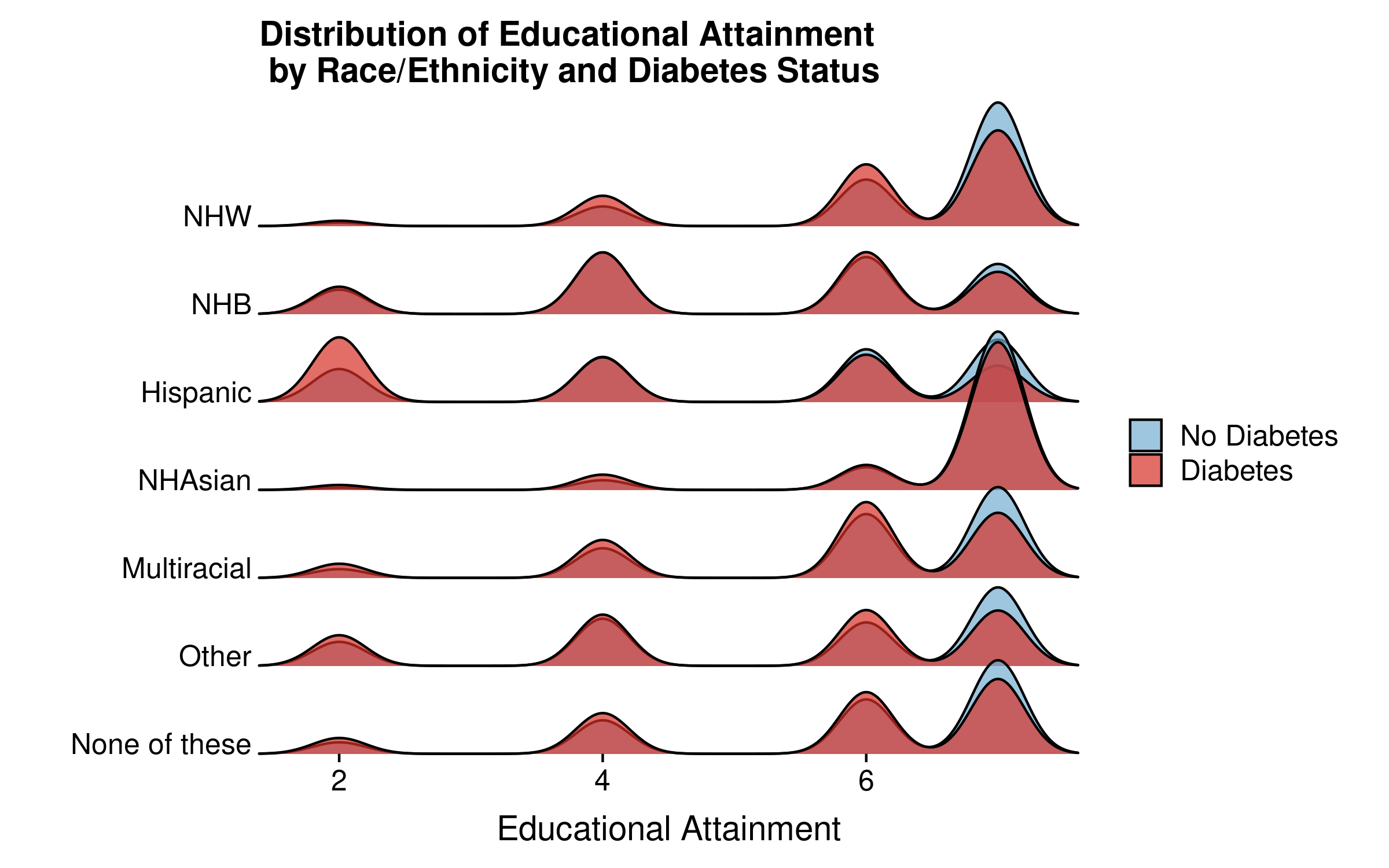

Supplement: S3 Fig — (PNG) [file pone.0351075.s008.png]

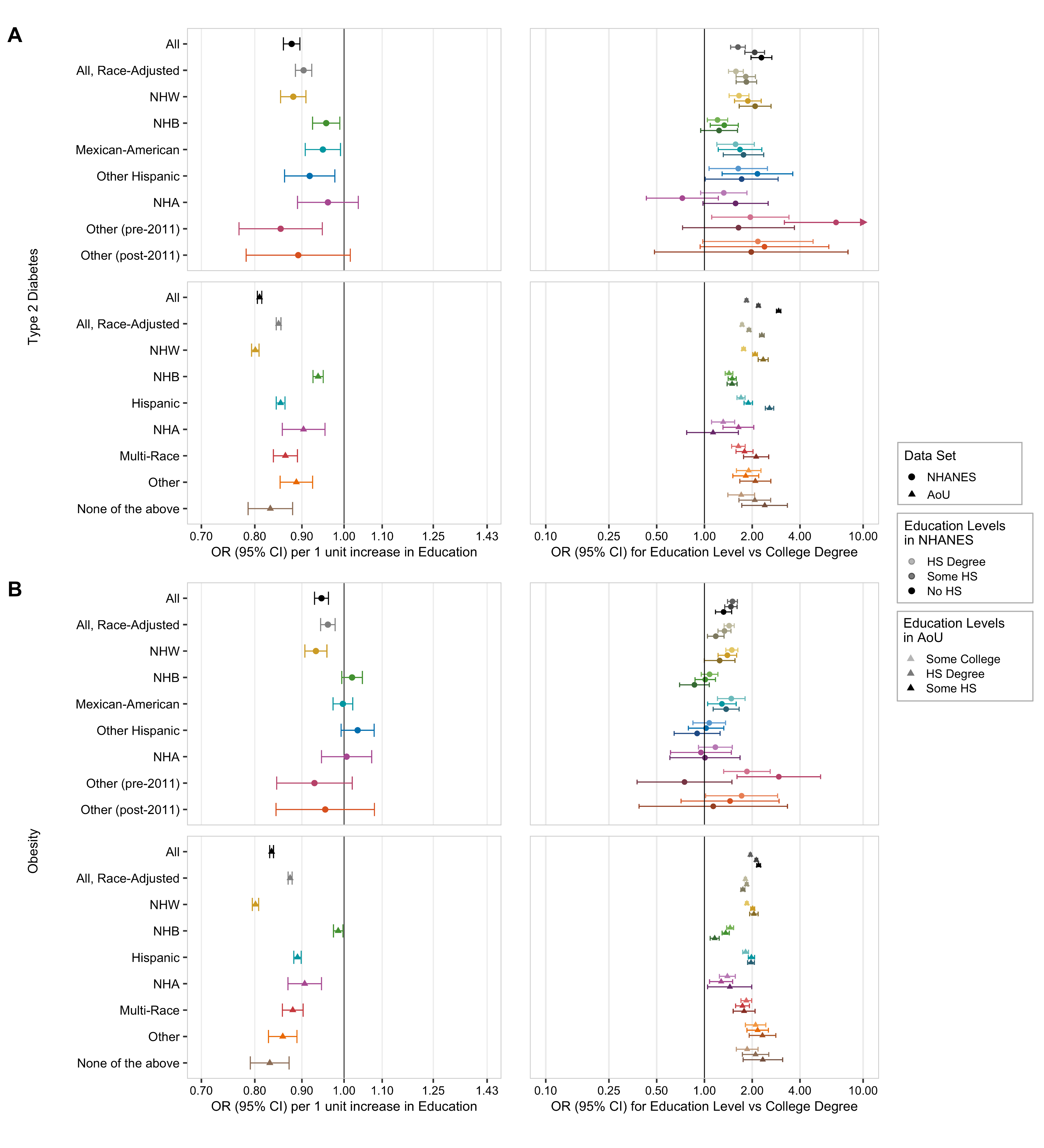

Supplement: S4 Fig — (TIFF) [file pone.0351075.s009.tiff]

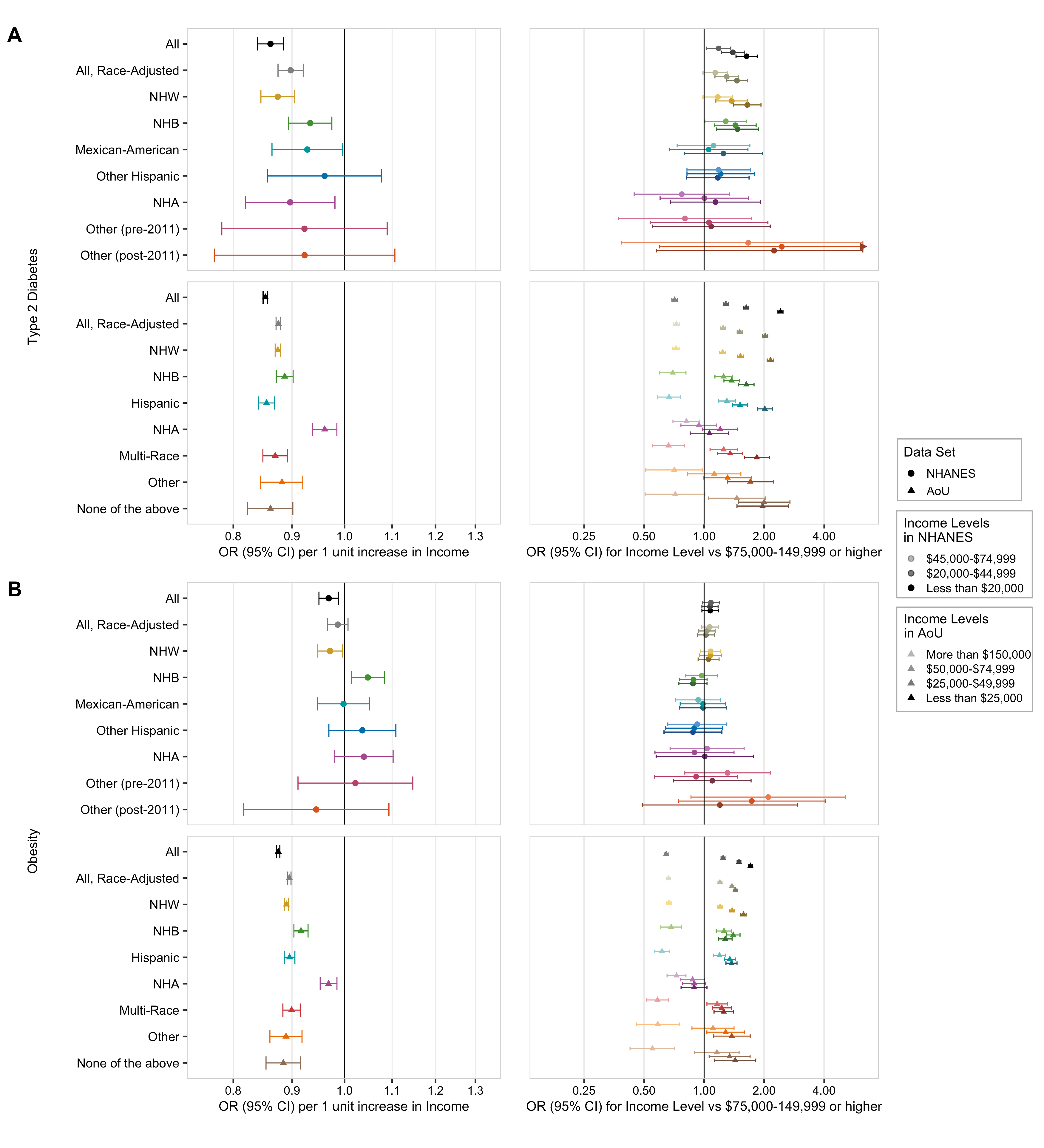

Supplement: S5 Fig — (TIFF) [file pone.0351075.s010.tiff]

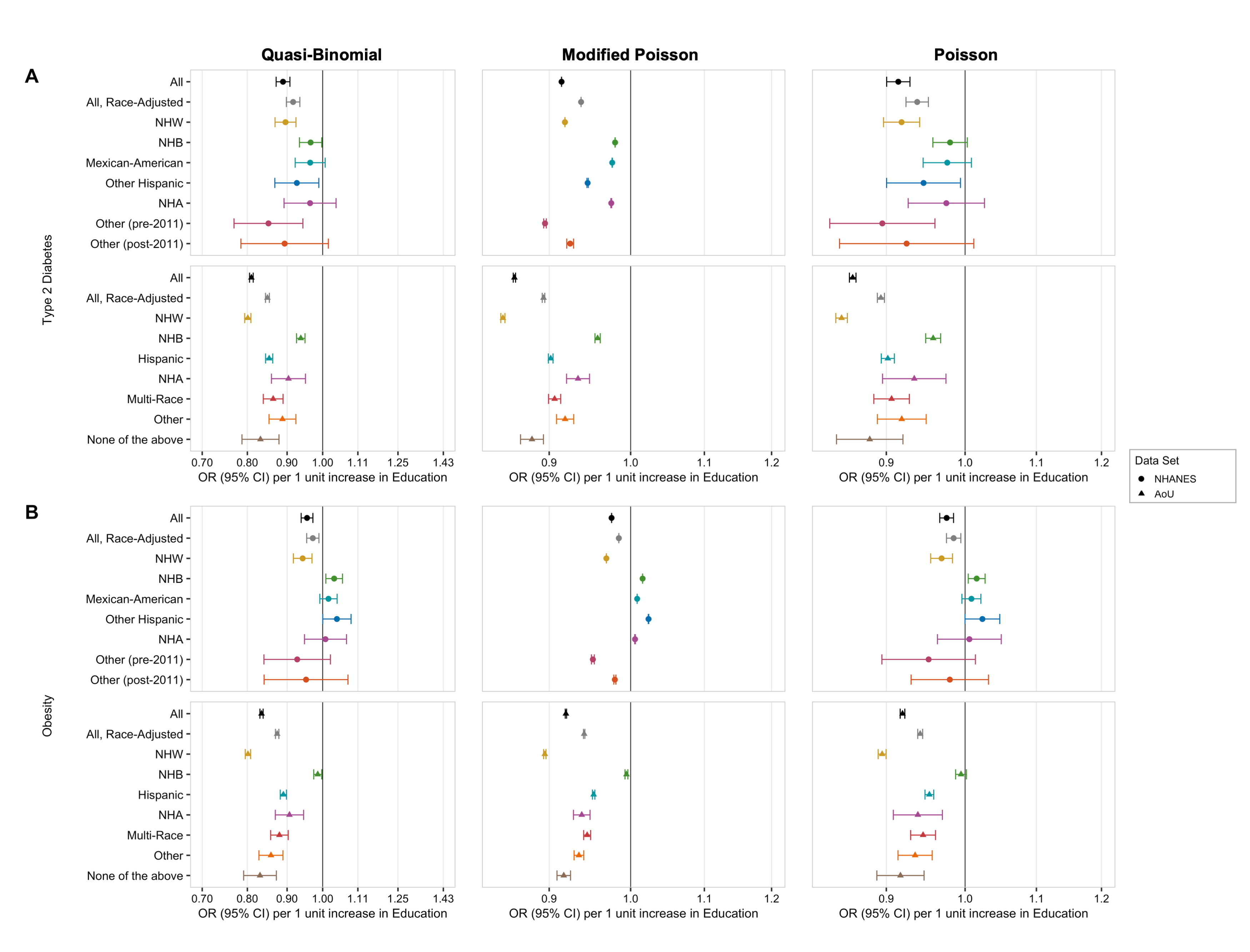

Supplement: S6 Fig — (TIFF) [file pone.0351075.s011.tiff]

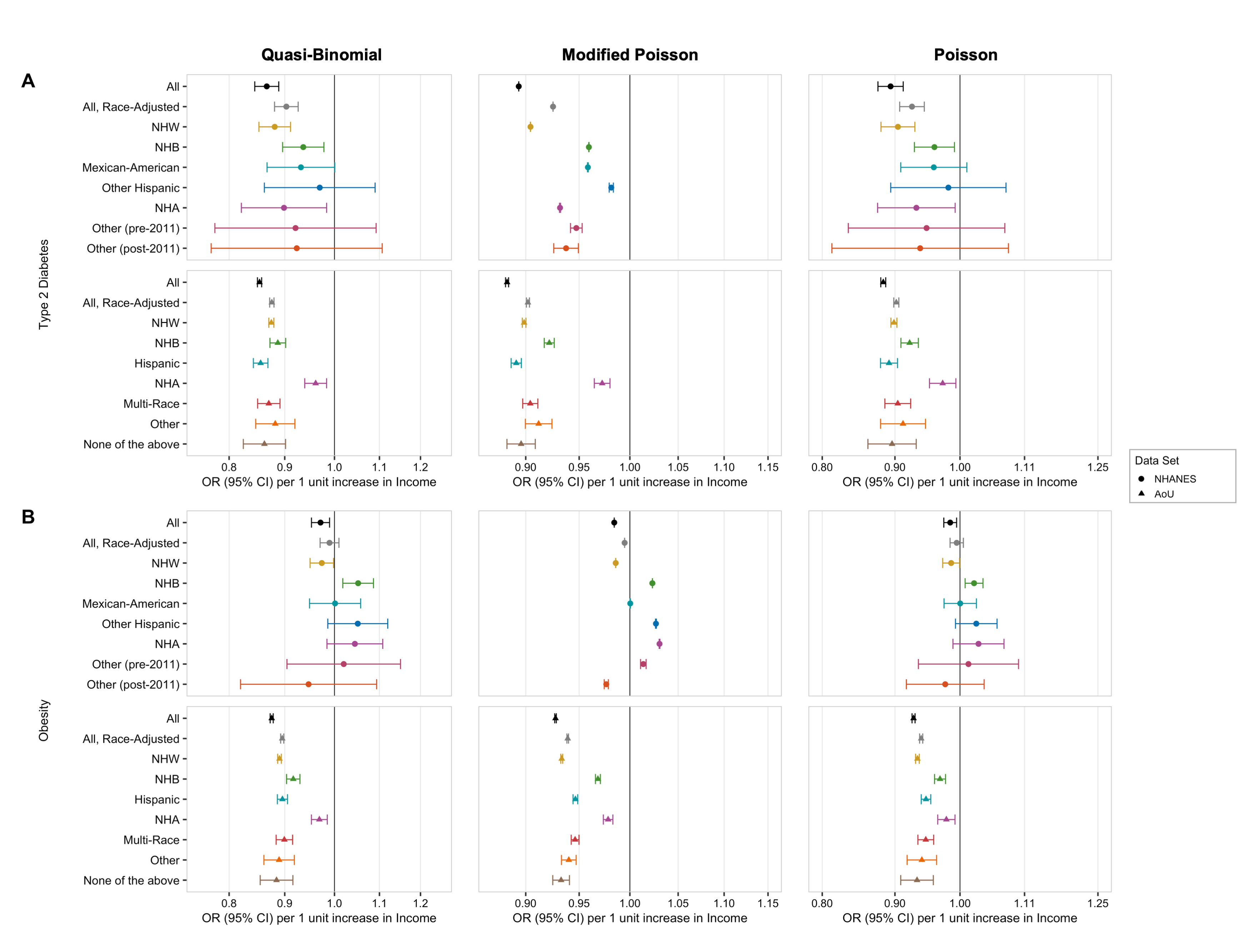

Supplement: S7 Fig — (TIFF) [file pone.0351075.s012.tiff]
