# Supplementary material for: Heterogeneous associations of socioeconomic status with metabolic disease in racial and ethnic subgroups in the United States: A cross-sectional cohort study in NHANES and All Of Us
Source: PLoS One. 2026 Jul 8;21(7):e0351075. doi: 10.1371/journal.pone.0351075 (PMC13345235; doi:10.1371/journal.pone.0351075)
Supplement: Attachment — Submitted filename: NHANES_AoU_RExSES_PLOSOne_response_to_reveiw_FINAL.docx [file pone.0351075.s013.docx]

Dear Dr. Azadnajafabad,

Thank you for the opportunity to revise our manuscript. Please find below a point-by-point discussion of the revisions we have made based on your and the reviewers’ recommendations. We appreciate your ongoing consideration.

Sincerely,

Sara Jane Cromer, MD, MS
50 Staniford St, Ste 340
Boston, MA 02114
617-726-8722
[scromer@mgh.harvard.edu](mailto:scromer@mgh.harvard.edu)

**Editor:**

1. Please ensure that your manuscript meets PLOS ONE's style requirements, including those for file naming. The PLOS ONE style templates can be found at <https://journals.plos.org/plosone/s/file?id=wjVg/PLOSOne_formatting_sample_main_body.pdf> and <https://journals.plos.org/plosone/s/file?id=ba62/PLOSOne_formatting_sample_title_authors_affiliations.pdf>
   a. We have ensured that formatting has been updated, including file names.
2. We note that the grant information you provided in the ‘Funding Information’ and ‘Financial Disclosure’ sections do not match. When you resubmit, please ensure that you provide the correct grant numbers for the awards you received for your study in the ‘Funding Information’ section.
   a. We have corrected this discrepancy. We had previously included only the PI for these grants in the “Funding Information” section based on the phrasing of the question (“Grant recipient”), but we now list all authors supported by each grant.
3. Thank you for stating the following financial disclosure: [SJC was supported by the American Diabetes Association (grant number 7-21-JDFM-005). CJP and SJC were supported by the National Institute of Environmental Health Sciences (grant number R01ES032470) and National Institutes of Diabetes, Digestive, and Kidney Diseases (grant number R01DK137993).].
   Please state what role the funders took in the study. If the funders had no role, please state: ""The funders had no role in study design, data collection and analysis, decision to publish, or preparation of the manuscript.""
   If this statement is not correct you must amend it as needed.
   Please include this amended Role of Funder statement in your cover letter; we will change the online submission form on your behalf.
   a. The suggested statement is correct, and we have added it to the cover letter.
4. Please note that your Data Availability Statement is currently missing the DOI/accession number of each dataset OR a direct link to access each database]. If your manuscript is accepted for publication, you will be asked to provide these details on a very short timeline. We therefore suggest that you provide this information now, though we will not hold up the peer review process if you are unable.
   a. We had previously included direct links to each dataset (NHANES, AoU) in our data availability statement:
      *Data availability statement: NHANES data is publicly available from the CDC website (https://www.cdc.gov/nchs/nhanes/index.htm) and should be handled using best practices outlined by NCHS. All of Us controlled-tier data is available by application to the All of Us Researcher Workbench (workbench.researchallofus.org).*
5. Please include captions for your Supporting Information files at the end of your manuscript, and update any in-text citations to match accordingly. Please see our Supporting Information guidelines for more information: <http://journals.plos.org/plosone/s/supporting-information>.
   a. This has been added after the citations as recommended.
6. If the reviewer comments include a recommendation to cite specific previously published works, please review and evaluate these publications to determine whether they are relevant and should be cited. There is no requirement to cite these works unless the editor has indicated otherwise.
   a. Thank you for this advice. The first reviewer recommended expanding our discussion of marginalization-related diminished returns, a field championed by Dr. Shivan Assari. We believe this is appropriate given the field’s relevance to our manuscript but had already cited multiple relevant papers by Dr. Assari, so our response has only modestly increased these citations.

Additional Editor Comments:

Authors may explain the reason to restrict the continuous NHANES data (1999-2023) to only 1999-2018 in this study.

**Response**: We utilized continuous NHANES 1999-2018 for two reasons. First, this analysis began several years ago, at which time 2017-2018 was the most recent cycle available (as you may know, updated NHANES data was not available for some time after the pandemic). Second, as we have revised this analysis over time, we have chosen not to use more recent cycles due to data collection disruption and irregularities which occurred due to the pandemic. While the overall sample has been re-weighted as a 2017-March 2020 cycle and is believed to be representative at a population-level, there are concerns that it may not be representative among subgroups. In particular, the NCHS notes:

*Trend comparisons for subgroups (for example, by age, sex, race and Hispanic origin) between the 2017–March 2020 prepandemic file and previous NHANES cycles should be interpreted with caution. The magnitude and direction of the differences between two cycles may vary by subgroup. This could cause changes in the magnitude or the direction of the trend within a certain subgroup, relative to overall changes. When conducting analyses and interpreting results, analysts should consider the historical context of the trends in addition to the methodological approach to create the 2017–March 2020 prepandemic file. (*[*https://www.cdc.gov/nchs/hus/sources-definitions/nhanes.htm*](https://www.cdc.gov/nchs/hus/sources-definitions/nhanes.htm)*, accessed 4/15/2026)*

Given the focus of our analysis on racial and ethnic subgroups, we did not want to introduce potential error by including this cycle, and therefore have not included the following cycle, as well. We have added this discussion to the limitations.
**Revised text** (Page 25): Our NHANES sample terminated in 2018 due to irregularities in field operations during the 2019-2020 cycle related to the COVID-19 pandemic; although the sample has been combined with the previous sample and re-weighted to create an overall nationally representative sample, there are concerns that this sample may not be representative within population subgroups, including racial and ethnic subgroups relevant to this work.27,62

Also, there is an updated release of All of Us Research Program data version 8 that could be used for this study.

**Response**: AoU v7 was the most up to date at the time of our analysis. We have updated the analysis to v8 which increased the sample size to 404,990 individuals. As expected, there were some small changes in exact estimates, but these were consistent with prior reported associations and continue to demonstrate different effects across racial and ethnic populations.

Regarding complex survey design and weights of NHANES, authors may add more details about the used weights (fasting and MEC weights in the case of this study) to make the results nationally representative.

**Response**: We have provided more details in the methods about NHANES weighting procedures. Of note, while fasting blood glucose, when available, was allowed to contribute to the type 2 diabetes case definition (impacted <7% of cases), no variables from the fasting sample were included in summary tables or regression analyses, so fasting weights were not used (only MEC weights)
**Revised text** (Page 8): We conducted all NHANES analyses (including summary of baseline characteristics and all regression models) using weighting procedures to account for complex survey design, as recommended by the NHANES statistical guidance documents.26 As the NHANES employs a multi-stage sampling approach, this including accounting for primary sampling units (PSU), sub-secondary sampling units, and individual-level exam weights to account for oversampling of sub-populations which varied by survey cycle.26,27

Regarding the logistic regression models, it is essential to mention if the models were survey-weighted too.

**Response**: All models used NHANES/NCHS-recommended survey weighting, and we have clarified this in the methods. Please see updated text above.

Regarding Tables 1 and 2, there are discrepancies on NHANES 2011-2018 column headers and the summarized data for 1999-2018.

**Response**: Thank you for noticing this – these headers have all been corrected to 1999-2018. We have also corrected this in Supplemental Figure 1 (NHANES flow diagram).

Regarding the wide period of study data and the issue of income comparability, it’s needed to consider measures to adjust for inflation, as income levels across a decade might not be directly comparable.

**Response**: Thank you for this comment, which is predominantly relevant to the NHANES (AoU recruited over a more restricted time period). In the NHANES, our primary income variable is continuous income-to-poverty ratio, where the poverty limit changes each year based on wider economic trends such that this ratio should be comparable over time. This is a limitation for the stratified analyses however, and we have added this to the limitations.
**Revised text** (Page 25): In NHANES, the income-to-poverty measures have a maximum value of 5.0, treating all participants with the highest income levels as equal; however, this measure has the strength of being relatively comparable over time, incorporating broad economic changes and inflation, which is not true for categorical income.

**Reviewer 1:**

I would first like to congratulate the authors for investigating heterogeneity in socioeconomic status (SES). The paper is timely and well written, with strong conceptual framing, robust methodology, and a large sample size.

**Response**: We thank the reviewer for their review which has strengthened the framing around minoritization-related diminished returns.

The study applies the Minorities’ Diminished Returns (MDRs) framework (Assari, 2018) and shows that SES indicators are associated with protection against cardiometabolic disorders; however, these protective effects appear weaker for Black and Latino populations and strongest for White and Asian American groups. Below are several areas that may benefit from further development:

Disease-specific MDRs literature

MDRs have been documented across several cardiometabolic outcomes, including obesity, cardiovascular disease, metabolic conditions, and diabetes. Incorporating and discussing these disease-specific findings would strengthen the discussion by situating the current results within existing evidence and demonstrating replication across outcomes.

NHANES-based MDRs studies

Previous MDRs research has used NHANES data to demonstrate diminished health returns among Black, Latino, and Native American populations. Because the current study also uses NHANES, citing and discussing these prior analyses would provide an additional layer of study-specific support and contextual replication.

Beyond race/ethnicity: other marginalized identities

MDRs have also been reported for other marginalized groups, including sexual minorities (e.g., LGBT populations) and individuals facing marginalization based on nativity or immigration status. Expanding the discussion to acknowledge these broader patterns may help position MDRs as a more general framework related to structural marginalization rather than race/ethnicity alone.

**Response**: We have expanded our discussion of MDR, including a broader swath of Dr. Assari’s extensive work in this field, both in the introduction and the discussion. This includes incorporation of additional references related to cardiometabolic health and related to the NHANES. We have maintained our primary focus in the discussion on racial and ethnic marginalization given the content of our study, but we have included a brief discussion that this phenomenon is not related to racial marginalization.
**Revised text** (Page 4): However, this relationship has been less explored in contemporary subpopulations of the United States, although a growing body of work highlights “diminished returns” of favorable SES for cardiometabolic and other chronic diseases in marginalized populations,8–15 including groups with marginalization related to race, gender, sexual identity, and immigration status.
**Revised text** (Page 21): At the individual level, some authors in the field have articulated a theory of “ marginalization-related diminished returns” of improved SES among marginalized and immigrant populations 45. For example, intersectionality of race or ethnicity and SES, has been reported for childhood BMI10,11,46 asthma,47 heart disease,12 and overall comorbidity or chronic disease burden,13–15,48 49in a variety of cross-sectional and cohort studies with diminished benefits seen in marginalized groups which notably include ( racially and ethnically minoritized groups as in our study but also members of the LGBTQIA community, and immigrants in both the USA and other nations).49 For example, a study performed among individuals with osteoarthritis found that higher income was associated with reduced odds of being overweight or obese among White individuals but not among Black individuals,8 with a related study showing a similar interaction between educational attainment and sexual orientation on obesity, with LGBTQIA individuals experiencing reduced benefits from increasing SES compared to heterosexual individuals.9 Similarly, several studies in the NHANES noted that the beneficial effects of educational attainment and employment on cardiometabolic health were attenuated in NHB adults compared to NHW adults.50 and in men compared to women.51 Related work in the AoU cohort also suggests that both SES effects and race-by-SES interactions may have disease-specific patterns.52 These consistent findings provide a foundation through which the findings of our study exploring the intersectionality of SES and race or ethnicity on metabolic disease across diverse groups in large, nationally representative datasets may be interpreted. Importantly, they highlight that this phenomenon of marginalization-related diminished returns is not limited to race- or ethnicity-related marginalization and that it may be observed across diverse populations and diseases.

Mechanisms underlying MDRs

MDRs should not be interpreted as a “magical” or unexplained process. A growing literature has identified potential mechanisms. For example, high-SES Black individuals may show weaker improvements in health behaviors such as physical activity and fruit and vegetable intake, higher exposure to stress and discrimination, elevated depressive symptoms, lower returns in income and pay despite education, poorer job quality, and continued exposure to disadvantaged neighborhood environments. These factors are all known predictors of cardiometabolic risk and may help explain why cardiometabolic conditions remain elevated among high-SES Black and Latino populations despite socioeconomic gains. Discussing these mechanisms would strengthen the interpretive depth of the paper.

**Response**: We agree and have expanded our discussion to include a review of factors which may mediate these diminished returns.
**Revised text** (Page 22): Although the correlates of marginalization-related diminished returns likely vary by subpopulation, society, and disease state, several potential mechanisms have been identified. For example, while educational attainment is associated with decreased health-related social needs globally, studies have shown that this effect is diminished in Latino populations, where educational attainment is less protective against food insecurity, possibility related to differences in employment.57 Similarly, cultural differences in dietary patterns or lifestyle behaviors, effects of structural racism such as segregation resulting in different physical environment exposures (e.g., adverse toxin or pollutant exposures), and chronic stress related to the experience of discrimination may continue to affect marginalized groups, particularly non-Hispanic Black individuals, despite attainment of greater SES.6,18,58–63

**Reviewer 2:**

The purpose of this analysis was to examine the intersection of socioeconomic indicators and race/ethnicity on the prevalence of type 2 diabetes and obesity. This fills a gap in our understanding of how different measures of SES may be associated with these outcomes and how that effect may be heterogeneous by race and ethnicity in the US.

Overall, I found this to be a very interesting, well-written, and considered manuscript. I was particularly intrigued because it uses NHANES, an extremely valuable cross-sectional dataset, and All of Us, a newer dataset with a different sampling paradigm and data collection process.

**Response**: We thank the reviewer for their time, as well as their detailed and thoughtful comments which we believe have strengthened the manuscript.

Major concerns

p7, line 163 - while standardizing to the projected 2000 population has been done for many years, two additional censuses have occurred since then to reflect our national composition. I would have preferred to see a more recent reference population or justification for why more recent counts were unsuitable for this analysis.

**Response**: We agree that age-standardization to a more recent census population may be more representative of the current U.S. population. For example, 2010 is approximately the median of our NHANES cycles. However, CDC/NCHS resources continue to recommend age standardization to the 2000 U.S. population when working with NHANES (see: <https://www.cdc.gov/nchs/hus/sources-definitions/age-adjustment.htm>), and we aimed to use the data based on NCHS-recommended best practices to be consistent with CDC reporting. We use the same adjustment in AoU for consistency between cohorts. We have clarified this justification in the methods.
**Revised text** (Page 8): We calculated age-adjusted disease prevalence by standardizing, within each race and ethnicity category, to the projected U.S. population in 2000, consistent with National Center for Health Statistics practices in the NHANES.27

Figure 3 fairly clearly shows that stratified by income, in the NHANES data, the effect of different levels of income are rather consistent within categories of race/ethnicity whereas in AoU, we see that “dose-response” also seen in the educational attainment analysis. I would like the authors to address this difference more thoroughly in the discussion and whether one data source should be weighted heavier in our interpretation than the other or whether both can be simultaneously true.

**Response**: Thank you for this comment, and we agree. We believe there are many factors that may explain the different patterns and magnitudes of association seen between datasets and means of transforming the SES measures, including selection bias in AoU which may even be population-specific (e.g., more than 40% of Asian individuals in AoU have an advanced degree, and 80% have at least a college degree) and subtle differences in variable transformation. We have substantially expanded this section of the discussion to acknowledge these nuances and allow the reader to consider these factors when interpreting study findings.
**Revised text** (Page 23-24): While we consistently find heterogeneous associations of SES with disease across race and ethnicity groups, our findings also revealed not only differences in magnitude and direction of SES effects on health but also differences in the pattern or shape of the association between these factors and disease in different data sets. This further illustrates the complexity of SES-disease modeling, whereby differences in dataset composition and selection bias, SES measure choice, and seemingly insignificant differences in variable transformation may lead to distinct patterns and magnitudes of association between SES and disease. For example, the magnitude and sometimes even direction of effect of the association with SES and disease differed in Asian populations in NHANES and AoU. We believe this is at least in part to selection bias in the AoU cohort, influencing both enrollment and data completeness in distinct patterns within race groups; for example, almost 80% of Asian AoU participants hold a college degree compared to approximately 50% in NHANES. As such, findings in NHANES, which allows for a nationally representative sample, may more closely describe true relationships within unselected Asian populations. Next, we see that different measures of SES – education and income – have different associations with disease outcomes, suggesting that the choice of socioeconomic measure should be carefully considered and interpreted, and if possible, standardized across research studies to improve interpretability and reproducibility of results. Finally, we see that differences in variable transformation meaningfully impact results. For example, in Figure 3 comparing the impact of income on disease across race and ethnicity groups, we observe differences (1) when SES is modeling continuously vs categorically, and (2) based on the means of categorizing income. Subtle differences in how income was transformed within datasets may contribute to these associations. For example, NHANES creates a continuous income-to-poverty ratio variable which has the advantage of incorporating household income, household size, and secular trends such as inflation to capture income in a more nuanced fashion. While we attempted to imitate this in AoU, transforming income to a continuous variable relative to the poverty limit in 2023, there are still subtle differences from NHANES. Most importantly, NHANES transforms all individuals with income-to-poverty ratio >5.0 as 5.0, creating an inability to capture variability within higher income groups. In categorical analyses, the income categories vary slightly between the two datasets, with an additional high income category that is not available in NHANES. Lastly, while our measures were all absolute, other studies may model SES relatively within a population (e.g., increase in disease risk per SD change in an area-level SES measure); when modeling within subpopulations, this may lead to incomparable results between groups due to differences in underlying SES distribution. For all of these reasons, the use of more than one socioeconomic measure, when available, and carefully planned sensitivity analyses to examine the impact of variable transformation and modeling on study results may better capture diverse elements of SES, improve rigor and reproducibility of SES-related analysis, and have greater predictive value for health outcomes, as seen in previous studies.65,66

Minor concerns

p21, line 423 - here you assert that educational attainment predates T2D onset; however, with the concerning prevalence of obesity among children, I fear that this is a weak argument without support evidence [here I acknowledge my own ignorance of T2D prevalence, but knowing its tie to obesity and the rates of obesity in children, I felt obligated to point out where your argument needs bolstering]

**Response**: We agree that both obesity and type 2 diabetes prevalence is growing among American youth. However, young people still make up a small fraction of people with type 2 diabetes (<https://gis.cdc.gov/grasp/diabetes/diabetesatlas-statsreport.html>). Additionally, given the difficulty of ascertaining type 1 vs. type 2 diabetes status in observational data, individuals with diabetes onset at age <30 were deemed to have type 1 diabetes in our datasets and not included in the type 2 diabetes cases. Given this, we believe it is appropriate to argue that educational attainment occurred before type 2 diabetes onset in the majority of individuals in our datasets. We have clarified this in the indicated section of the limitations, and also softened this statement.
**Revised text** (Page 25): however, the age at which participants attain their SES, especially educational attainment, is most likely to come before T2D onset and study entry (particularly as our population included only adults, and T2D cases must have had diabetes onset after age 30), making reverse causality less likely.

p22, lines 438 - 442 - you list a lot of different factors that could play into why the results we see in NHANES and AoU may be different, but you give no indication on how they could have affected result estimates or if any of those differences makes you more or less confident in the results.

**Response**: We have expanded this section to explore the between-population heterogeneity, specifically for the NHA group where the selection bias was most striking. Although there are modest differences between these groups, confidence intervals were overlapping and directionality of effects were consistent.
**Revised text** (Page 26): For example, the NHA cohort in AoU had very high levels of educational attainment and income, suggesting a selected population which is not representative of the US population and demonstrated a different pattern of association between SES and metabolic disease, albeit with overlapping confidence intervals. However, the consistent directionality of effects, which remain distinct from the NHW population in both datasets, suggests that our primary findings of heterogeneous effects of SES across racial and ethnic groups are robust even in the face of significant selection bias and that these patterns persist even in high-SES groups.
