# Supplementary material for: Heterogeneous associations of socioeconomic status with metabolic disease in racial and ethnic subgroups in the United States: A cross-sectional cohort study in NHANES and All Of Us
Source: PLoS One. 2026 Jul 8;21(7):e0351075. doi: 10.1371/journal.pone.0351075 (PMC13345235; doi:10.1371/journal.pone.0351075)
Supplement: Peer review history

# Peer review: Decision Letter 0

**DOI:** 10.1371/journal.pone.0351075.r001

**Authors:** Sina Azadnajafabad

13 Apr 2026

PONE-D-25-65931

Heterogeneous Associations of Socioeconomic Status with Metabolic Disease in Racial and Ethnic Subgroups in the United States: A Cross-Sectional Cohort Study in NHANES and All Of Us

PLOS One

Dear Dr. Cromer,

Thank you for submitting your manuscript to PLOS ONE. After careful consideration, we feel that it has merit but does not fully meet PLOS ONE’s publication criteria as it currently stands. Therefore, we invite you to submit a revised version of the manuscript that addresses the points raised during the review process.

Please submit your revised manuscript by May 28 2026 11:59PM. If you will need more time than this to complete your revisions, please reply to this message or contact the journal office at plosone@plos.org. When you're ready to submit your revision, log on to https://www.editorialmanager.com/pone/ and select the 'Submissions Needing Revision' folder to locate your manuscript file.

Please include the following items when submitting your revised manuscript:

- A letter that responds to each point raised by the academic editor and reviewer(s). You should upload this letter as a separate file labeled 'Response to Reviewers'.
- A marked-up copy of your manuscript that highlights changes made to the original version. You should upload this as a separate file labeled 'Revised Manuscript with Track Changes'.
- An unmarked version of your revised paper without tracked changes. You should upload this as a separate file labeled 'Manuscript'.

If you would like to make changes to your financial disclosure, please include your updated statement in your cover letter. Guidelines for resubmitting your figure files are available below the reviewer comments at the end of this letter.

If applicable, we recommend that you deposit your laboratory protocols in protocols.io to enhance the reproducibility of your results. Protocols.io assigns your protocol its own identifier (DOI) so that it can be cited independently in the future. For instructions see: https://journals.plos.org/plosone/s/submission-guidelines#loc-laboratory-protocols. Additionally, PLOS ONE offers an option for publishing peer-reviewed Lab Protocol articles, which describe protocols hosted on protocols.io. Read more information on sharing protocols at https://plos.org/protocols?utm_medium=editorial-email&utm_source=authorletters&utm_campaign=protocols.

As the corresponding author, your ORCID iD is verified in the submission system and will appear in the published article. PLOS supports the use of ORCID, and we encourage all coauthors to register for an ORCID iD and use it as well. Please encourage your coauthors to verify their ORCID iD within the submission system before final acceptance, as unverified ORCID iDs will not appear in the published article. *Only*    the individual author can complete the verification step; PLOS staff *cannot*    verify ORCID iDs on behalf of authors.

We look forward to receiving your revised manuscript.

Kind regards,

Sina Azadnajafabad, MD, MPH

Academic Editor

PLOS One

Journal Requirements:

When submitting your revision, we need you to address these additional requirements.

1. Please ensure that your manuscript meets PLOS ONE's style requirements, including those for file naming. The PLOS ONE style templates can be found at

https://journals.plos.org/plosone/s/file?id=wjVg/PLOSOne_formatting_sample_main_body.pdf and

https://journals.plos.org/plosone/s/file?id=ba62/PLOSOne_formatting_sample_title_authors_affiliations.pdf

2. We note that the grant information you provided in the ‘Funding Information’ and ‘Financial Disclosure’ sections do not match.

When you resubmit, please ensure that you provide the correct grant numbers for the awards you received for your study in the ‘Funding Information’ section.

3. Thank you for stating the following financial disclosure: [SJC was supported by the American Diabetes Association (grant number 7-21-JDFM-005). CJP and SJC were supported by the National Institute of Environmental Health Sciences (grant number R01ES032470) and National Institutes of Diabetes, Digestive, and Kidney Diseases (grant number R01DK137993).].

Please state what role the funders took in the study.  If the funders had no role, please state: ""The funders had no role in study design, data collection and analysis, decision to publish, or preparation of the manuscript.""

If this statement is not correct you must amend it as needed.

Please include this amended Role of Funder statement in your cover letter; we will change the online submission form on your behalf.

4. Please note that your Data Availability Statement is currently missing the DOI/accession number of each dataset OR a direct link to access each database]. If your manuscript is accepted for publication, you will be asked to provide these details on a very short timeline. We therefore suggest that you provide this information now, though we will not hold up the peer review process if you are unable.

5. Please include captions for your Supporting Information files at the end of your manuscript, and update any in-text citations to match accordingly. Please see our Supporting Information guidelines for more information: http://journals.plos.org/plosone/s/supporting-information.

6. If the reviewer comments include a recommendation to cite specific previously published works, please review and evaluate these publications to determine whether they are relevant and should be cited. There is no requirement to cite these works unless the editor has indicated otherwise.

Additional Editor Comments:

Authors may explain the reason to restrict the continuous NHANES data (1999-2023) to only 1999-2018 in this study. Also, there is an updated release of All of Us Research Program data version 8 that could be used for this study. Regarding complex survey design and weights of NHANES, authors may add more details about the used weights (fasting and MEC weights in the case of this study) to make the results nationally representative. Regarding the logistic regression models, it is essential to mention if the models were survey-weighted too. Regarding Tables 1 and 2, there are discrepancies on NHANES 2011-2018 column headers and the summarized data for 1999-2018. Regarding the wide period of study data and the issue of income comparability, it’s needed to consider measures to adjust for inflation, as income levels across a decade might not be directly comparable.

[Note: HTML markup is below. Please do not edit.]

Reviewers' comments:

Reviewer's Responses to Questions

**Comments to the Author**

1. Is the manuscript technically sound, and do the data support the conclusions?

The manuscript must describe a technically sound piece of scientific research with data that supports the conclusions. Experiments must have been conducted rigorously, with appropriate controls, replication, and sample sizes. The conclusions must be drawn appropriately based on the data presented.

Reviewer #1: Yes

Reviewer #2: Yes

**********

2. Has the statistical analysis been performed appropriately and rigorously?

Reviewer #1: Yes

Reviewer #2: Yes

**********

3. Have the authors made all data underlying the findings in their manuscript fully available?

The PLOS Data policy requires authors to make all data underlying the findings described in their manuscript fully available without restriction, with rare exception (please refer to the Data Availability Statement in the manuscript PDF file). The data should be provided as part of the manuscript or its supporting information, or deposited to a public repository. For example, in addition to summary statistics, the data points behind means, medians and variance measures should be available. If there are restrictions on publicly sharing data—e.g. participant privacy or use of data from a third party—those must be specified.

Reviewer #1: Yes

Reviewer #2: Yes

**********

4. Is the manuscript presented in an intelligible fashion and written in standard English?

PLOS ONE does not copyedit accepted manuscripts, so the language in submitted articles must be clear, correct, and unambiguous. Any typographical or grammatical errors should be corrected at revision, so please note any specific errors here.

Reviewer #1: Yes

Reviewer #2: Yes

**********

5. Review Comments to the Author

Please use the space provided to explain your answers to the questions above. You may also include additional comments for the author, including concerns about dual publication, research ethics, or publication ethics. (Please upload your review as an attachment if it exceeds 20,000 characters)

Reviewer #1: I would first like to congratulate the authors for investigating heterogeneity in socioeconomic status (SES). The paper is timely and well written, with strong conceptual framing, robust methodology, and a large sample size. The study applies the Minorities’ Diminished Returns (MDRs) framework (Assari, 2018) and shows that SES indicators are associated with protection against cardiometabolic disorders; however, these protective effects appear weaker for Black and Latino populations and strongest for White and Asian American groups. Below are several areas that may benefit from further development:

Disease-specific MDRs literature

MDRs have been documented across several cardiometabolic outcomes, including obesity, cardiovascular disease, metabolic conditions, and diabetes. Incorporating and discussing these disease-specific findings would strengthen the discussion by situating the current results within existing evidence and demonstrating replication across outcomes.

NHANES-based MDRs studies

Previous MDRs research has used NHANES data to demonstrate diminished health returns among Black, Latino, and Native American populations. Because the current study also uses NHANES, citing and discussing these prior analyses would provide an additional layer of study-specific support and contextual replication.

Beyond race/ethnicity: other marginalized identities

MDRs have also been reported for other marginalized groups, including sexual minorities (e.g., LGBT populations) and individuals facing marginalization based on nativity or immigration status. Expanding the discussion to acknowledge these broader patterns may help position MDRs as a more general framework related to structural marginalization rather than race/ethnicity alone.

Mechanisms underlying MDRs

MDRs should not be interpreted as a “magical” or unexplained process. A growing literature has identified potential mechanisms. For example, high-SES Black individuals may show weaker improvements in health behaviors such as physical activity and fruit and vegetable intake, higher exposure to stress and discrimination, elevated depressive symptoms, lower returns in income and pay despite education, poorer job quality, and continued exposure to disadvantaged neighborhood environments. These factors are all known predictors of cardiometabolic risk and may help explain why cardiometabolic conditions remain elevated among high-SES Black and Latino populations despite socioeconomic gains. Discussing these mechanisms would strengthen the interpretive depth of the paper.

Reviewer #2: The purpose of this analysis was to examine the intersection of socioeconomic indicators and race/ethnicity on the prevalence of type 2 diabetes and obesity. This fills a gap in our understanding of how different measures of SES may be associated with these outcomes and how that effect may be heterogeneous by race and ethnicity in the US.

Overall, I found this to be a very interesting, well-written, and considered manuscript. I was particularly intrigued because it uses NHANES, an extremely valuable cross-sectional dataset, and All of Us, a newer dataset with a different sampling paradigm and data collection process.

Major concerns

p7, line 163 - while standardizing to the projected 2000 population has been done for many years, two additional censuses have occurred since then to reflect our national composition. I would have preferred to see a more recent reference population or justification for why more recent counts were unsuitable for this analysis.

Figure 3 fairly clearly shows that stratified by income, in the NHANES data, the effect of different levels of income are rather consistent within categories of race/ethnicity whereas in AoU, we see that “dose-response” also seen in the educational attainment analysis. I would like the authors to address this difference more thoroughly in the discussion and whether one data source should be weighted heavier in our interpretation than the other or whether both can be simultaneously true.

Minor concerns

p21, line 423 - here you assert that educational attainment predates T2D onset; however, with the concerning prevalence of obesity among children, I fear that this is a weak argument without support evidence [here I acknowledge my own ignorance of T2D prevalence, but knowing its tie to obesity and the rates of obesity in children, I felt obligated to point out where your argument needs bolstering]

p22, lines 438 - 442 - you list a lot of different factors that could play into why the results we see in NHANES and AoU may be different, but you give no indication on how they could have affected result estimates or if any of those differences makes you more or less confident in the results.

**********

6. PLOS authors have the option to publish the peer review history of their article (what does this mean?). If published, this will include your full peer review and any attached files.

If you choose “no”, your identity will remain anonymous but your review may still be made public.

**Do you want your identity to be public for this peer review?**    For information about this choice, including consent withdrawal, please see our Privacy Policy.

Reviewer #1: No

Reviewer #2: **Yes:**    Lauren Parlett

**********

[NOTE: If reviewer comments were submitted as an attachment file, they will be attached to this email and accessible via the submission site. Please log into your account, locate the manuscript record, and check for the action link "View Attachments". If this link does not appear, there are no attachment files.]

To ensure your figures meet our technical requirements, please review our figure guidelines: https://journals.plos.org/plosone/s/figures

You may also use PLOS’s free figure tool, NAAS, to help you prepare publication quality figures: https://journals.plos.org/plosone/s/figures#loc-tools-for-figure-preparation.

NAAS will assess whether your figures meet our technical requirements by comparing each figure against our figure specifications.

# Author comment: Author response to Decision Letter 1

**DOI:** 10.1371/journal.pone.0351075.r002

7 May 2026

Dear Dr. Azadnajafabad,

Thank you for the opportunity to revise our manuscript. Please find below a point-by-point discussion of the revisions we have made based on your and the reviewers’ recommendations. We appreciate your ongoing consideration.

Sincerely,

Sara Jane Cromer, MD, MS

50 Staniford St, Ste 340

Boston, MA 02114

617-726-8722

scromer@mgh.harvard.edu

Editor:

1. Please ensure that your manuscript meets PLOS ONE's style requirements, including those for file naming. The PLOS ONE style templates can be found at https://journals.plos.org/plosone/s/file?id=wjVg/PLOSOne_formatting_sample_main_body.pdf and https://journals.plos.org/plosone/s/file?id=ba62/PLOSOne_formatting_sample_title_authors_affiliations.pdf

a. We have ensured that formatting has been updated, including file names.

2. We note that the grant information you provided in the ‘Funding Information’ and ‘Financial Disclosure’ sections do not match. When you resubmit, please ensure that you provide the correct grant numbers for the awards you received for your study in the ‘Funding Information’ section.

a. We have corrected this discrepancy. We had previously included only the PI for these grants in the “Funding Information” section based on the phrasing of the question (“Grant recipient”), but we now list all authors supported by each grant.

3. Thank you for stating the following financial disclosure: [SJC was supported by the American Diabetes Association (grant number 7-21-JDFM-005). CJP and SJC were supported by the National Institute of Environmental Health Sciences (grant number R01ES032470) and National Institutes of Diabetes, Digestive, and Kidney Diseases (grant number R01DK137993).].

Please state what role the funders took in the study. If the funders had no role, please state: ""The funders had no role in study design, data collection and analysis, decision to publish, or preparation of the manuscript.""

If this statement is not correct you must amend it as needed.

Please include this amended Role of Funder statement in your cover letter; we will change the online submission form on your behalf.

a. The suggested statement is correct, and we have added it to the cover letter.

4. Please note that your Data Availability Statement is currently missing the DOI/accession number of each dataset OR a direct link to access each database]. If your manuscript is accepted for publication, you will be asked to provide these details on a very short timeline. We therefore suggest that you provide this information now, though we will not hold up the peer review process if you are unable.

a. We had previously included direct links to each dataset (NHANES, AoU) in our data availability statement:

Data availability statement: NHANES data is publicly available from the CDC website (https://www.cdc.gov/nchs/nhanes/index.htm) and should be handled using best practices outlined by NCHS. All of Us controlled-tier data is available by application to the All of Us Researcher Workbench (workbench.researchallofus.org).

5. Please include captions for your Supporting Information files at the end of your manuscript, and update any in-text citations to match accordingly. Please see our Supporting Information guidelines for more information: http://journals.plos.org/plosone/s/supporting-information.

a. This has been added after the citations as recommended.

6. If the reviewer comments include a recommendation to cite specific previously published works, please review and evaluate these publications to determine whether they are relevant and should be cited. There is no requirement to cite these works unless the editor has indicated otherwise.

a. Thank you for this advice. The first reviewer recommended expanding our discussion of marginalization-related diminished returns, a field championed by Dr. Shivan Assari. We believe this is appropriate given the field’s relevance to our manuscript but had already cited multiple relevant papers by Dr. Assari, so our response has only modestly increased these citations.

Additional Editor Comments:

Authors may explain the reason to restrict the continuous NHANES data (1999-2023) to only 1999-2018 in this study.

Response: We utilized continuous NHANES 1999-2018 for two reasons. First, this analysis began several years ago, at which time 2017-2018 was the most recent cycle available (as you may know, updated NHANES data was not available for some time after the pandemic). Second, as we have revised this analysis over time, we have chosen not to use more recent cycles due to data collection disruption and irregularities which occurred due to the pandemic. While the overall sample has been re-weighted as a 2017-March 2020 cycle and is believed to be representative at a population-level, there are concerns that it may not be representative among subgroups. In particular, the NCHS notes:

Trend comparisons for subgroups (for example, by age, sex, race and Hispanic origin) between the 2017–March 2020 prepandemic file and previous NHANES cycles should be interpreted with caution. The magnitude and direction of the differences between two cycles may vary by subgroup. This could cause changes in the magnitude or the direction of the trend within a certain subgroup, relative to overall changes. When conducting analyses and interpreting results, analysts should consider the historical context of the trends in addition to the methodological approach to create the 2017–March 2020 prepandemic file. (https://www.cdc.gov/nchs/hus/sources-definitions/nhanes.htm, accessed 4/15/2026)

Given the focus of our analysis on racial and ethnic subgroups, we did not want to introduce potential error by including this cycle, and therefore have not included the following cycle, as well. We have added this discussion to the limitations.

Revised text (Page 25): Our NHANES sample terminated in 2018 due to irregularities in field operations during the 2019-2020 cycle related to the COVID-19 pandemic; although the sample has been combined with the previous sample and re-weighted to create an overall nationally representative sample, there are concerns that this sample may not be representative within population subgroups, including racial and ethnic subgroups relevant to this work.27,62

Also, there is an updated release of All of Us Research Program data version 8 that could be used for this study.

Response: AoU v7 was the most up to date at the time of our analysis. We have updated the analysis to v8 which increased the sample size to 404,990 individuals. As expected, there were some small changes in exact estimates, but these were consistent with prior reported associations and continue to demonstrate different effects across racial and ethnic populations.

Regarding complex survey design and weights of NHANES, authors may add more details about the used weights (fasting and MEC weights in the case of this study) to make the results nationally representative.

Response: We have provided more details in the methods about NHANES weighting procedures. Of note, while fasting blood glucose, when available, was allowed to contribute to the type 2 diabetes case definition (impacted <7% of cases), no variables from the fasting sample were included in summary tables or regression analyses, so fasting weights were not used (only MEC weights)

Revised text (Page 8): We conducted all NHANES analyses (including summary of baseline characteristics and all regression models) using weighting procedures to account for complex survey design, as recommended by the NHANES statistical guidance documents.26 As the NHANES employs a multi-stage sampling approach, this including accounting for primary sampling units (PSU), sub-secondary sampling units, and individual-level exam weights to account for oversampling of sub-populations which varied by survey cycle.26,27

Regarding the logistic regression models, it is essential to mention if the models were survey-weighted too.

Response: All models used NHANES/NCHS-recommended survey weighting, and we have clarified this in the methods. Please see updated text above.

Regarding Tables 1 and 2, there are discrepancies on NHANES 2011-2018 column headers and the summarized data for 1999-2018.

Response: Thank you for noticing this – these headers have all been corrected to 1999-2018. We have also corrected this in Supplemental Figure 1 (NHANES flow diagram).

Regarding the wide period of study data and the issue of income comparability, it’s needed to consider measures to adjust for inflation, as income levels across a decade might not be directly comparable.

Response: Thank you for this comment, which is predominantly relevant to the NHANES (AoU recruited over a more restricted time period). In the NHANES, our primary income variable is continuous income-to-poverty ratio, where the poverty limit changes each year based on wider economic trends such that this ratio should be comparable over time. This is a limitation for the stratified analyses however, and we have added this to the limitations.

Revised text (Page 25): In NHANES, the income-to-poverty measures have a maximum value of 5.0, treating all participants with the highest income levels as equal; however, this measure has the strength of being relatively comparable over time, incorporating broad economic changes and inflation, which is not true for categorical income.

Reviewer 1:

I would first like to congratulate the authors for investigating heterogeneity in socioeconomic status (SES). The paper is timely and well written, with strong conceptual framing, robust methodology, and a large sample size.

Response: We thank the reviewer for their review which has strengthened the framing around minoritization-related diminished returns.

The study applies the Minorities’ Diminished Returns (MDRs) framework (Assari, 2018) and shows that SES indicators are associated with protection against cardiometabolic disorders; however, these protective effects appear weaker for Black and Latino populations and strongest for White and Asian American groups. Below are several areas that may benefit from further development:

Disease-specific MDRs literature

MDRs have been documented across several cardiometabolic outcomes, including obesity, cardiovascular disease, metabolic conditions, and diabetes. Incorporating and discussing these disease-specific findings would strengthen the discussion by situating the current results within existing evidence and demonstrating replication across outcomes.

NHANES-based MDRs studies

Previous MDRs research has used NHANES data to demonstrate diminished health returns among Black, Latino, and Native American populations. Because the current study also uses NHANES, citing and discussing these prior analyses would provide an additional layer of study-specific support and contextual replication.

Beyond race/ethnicity: other marginalized identities

MDRs have also been reported for other marginalized groups, including sexual minorities (e.g., LGBT populations) and individuals facing marginalization based on nativity or immigration status. Expanding the discussion to acknowledge these broader patterns may help position MDRs as a more general framework related to structural marginalization rather than race/ethnicity alone.

Response: We have expanded our discussion of MDR, including a broader swath of Dr. Assari’s extensive work in this field, both in the introduction and the discussion. This includes incorporation of additional references related to cardiometabolic health and related to the NHANES. We have maintained our primary focus in the discussion on racial and ethnic marginalization given the content of our study, but we have included a brief discussion that this phenomenon is not related to racial marginalization.

Revised text (Page 4): However, this relationship has been less explored in contemporary subpopulations of the United States, although a growing body of work highlights “diminished returns” of favorable SES for cardiometabolic and other chronic diseases in marginalized populations,8–15 including groups with marginalization related to race, gender, sexual identity, and immigration status.

Revised text (Page 21): At the individual level, some authors in the field have articulated a theory of “ marginalization-related diminished returns” of improved SES among marginalized and immigrant populations 45. For example, intersectionality of race or ethnicity and SES, has been reported for childhood BMI10,11,46 asthma,47 heart disease,12 and overall comorbidity or chronic disease burden,13–15,48 49in a variety of cross-sectional and cohort studies with diminished benefits seen in marginalized groups which notably include ( racially and ethnically minoritized groups as in our study but also members of the LGBTQIA community, and immigrants in both the USA and other nations).49 For example, a study performed among individuals with osteoarthritis found that higher income was associated with reduced odds of being overweight or obese among White individuals but not among Black individuals,8 with a related study showing a similar interaction between educational attainment and sexual orientation on obesity, with LGBTQIA individuals experiencing reduced benefits from increasing SES compared to heterosexual individuals.9 Similarly, several studies in the NHANES noted that the beneficial effects of educational attainment and employment on cardiometabolic health were attenuated in NHB adults compared to NHW adults.50 and in men compared to women.51 Related work in the AoU cohort also suggests that both SES effects and race-by-SES interactions may have disease-specific patterns.52 These consistent findings provide a foundation through which the findings of our study exploring the intersectionality of SES and race or ethnicity on metabolic disease across diverse groups in large, nationally representative datasets may be interpreted. Importantly, they highlight that this phenomenon of marginalization-related diminished returns is not limited to race- or ethnicity-related marginalization and that it may be observed across diverse populations and diseases.

Mechanisms underlying MDRs

MDRs should not be interpreted as a “magical” or unexplained process. A growing literature has identified potential mechanisms. For example, high-SES Black individuals may show weaker improvements in health behaviors such as physical activity and fruit and vegetable intake, higher exposure to stress and discrimination, elevated depressive symptoms, lower returns in income and pay despite education, poorer job quality, and continued exposure to disadvantaged neighborhood environments. These factors are all known predictors of cardiometabolic risk and may help explain why cardiometabolic conditions remain elevated among high-SES Black and Latino populations despite socioeconomic gains. Discussing these mechanisms would strengthen the interpretive depth of the paper.

Response: We agree and have expanded our discussion to include a review of factors which may mediate these diminished returns.

Revised text (Page 22): Although the correlates of marginalization-related diminished returns likely vary by subpopulation, society, and disease state, several potential mechanisms have been identified. For example, while educational attainment is associated with decreased health-related social needs globally, studies have shown that this effect is diminished in Latino populations, where educational attainment is less protective against food insecurity, possibility related to differences in employment.57 Similarly, cultural differences in dietary patterns or lifestyle behaviors, effects of structural racism such as segregation resulting in different physical environment exposures (e.g., adverse toxin or pollutant exposures), and chronic stress related to the experience of discrimination may continue to affect marginalized groups, particularly non-Hispanic Black individuals, despite attainment of greater SES.6,18,58–63

Reviewer 2:

The purpose of this analysis was to examine the intersection of socioeconomic indicators and race/ethnicity on the prevalence of type 2 diabetes and obesity. This fills a gap in our understanding of how different measures of SES may be associated with these outcom

Attachment Submitted filename: NHANES_AoU_RExSES_PLOSOne_response_to_reveiw_FINAL.docx

# Peer review: Decision Letter 1

**DOI:** 10.1371/journal.pone.0351075.r003

**Authors:** Sina Azadnajafabad

22 May 2026

Heterogeneous Associations of Socioeconomic Status with Metabolic Disease in Racial and Ethnic Subgroups in the United States: A Cross-Sectional Cohort Study in NHANES and All Of Us

PONE-D-25-65931R1

Dear Dr. Cromer,

We’re pleased to inform you that your manuscript has been judged scientifically suitable for publication and will be formally accepted for publication once it meets all outstanding technical requirements.

Within one week, you’ll receive an e-mail detailing the required amendments. When these have been addressed, you’ll receive a formal acceptance letter and your manuscript will be scheduled for publication.

An invoice will be generated when your article is formally accepted. Please note, if your institution has a publishing partnership with PLOS and your article meets the relevant criteria, all or part of your publication costs will be covered. Please make sure your user information is up-to-date by logging into Editorial Manager at Editorial Manager® and clicking the ‘Update My Information' link at the top of the page. For questions related to billing, please contact billing support.

If your institution or institutions have a press office, please notify them about your upcoming paper to help maximize its impact. If they’ll be preparing press materials, please inform our press team as soon as possible -- no later than 48 hours after receiving the formal acceptance. Your manuscript will remain under strict press embargo until 2 pm Eastern Time on the date of publication. For more information, please contact onepress@plos.org.

Kind regards,

Sina Azadnajafabad, MD, MPH

Academic Editor

PLOS One

Additional Editor Comments (optional):

Reviewers' comments:

Reviewer's Responses to Questions

**Comments to the Author**

1. If the authors have adequately addressed your comments raised in a previous round of review and you feel that this manuscript is now acceptable for publication, you may indicate that here to bypass the “Comments to the Author” section, enter your conflict of interest statement in the “Confidential to Editor” section, and submit your "Accept" recommendation.

Reviewer #1: (No Response)

Reviewer #2: All comments have been addressed

**********

2. Is the manuscript technically sound, and do the data support the conclusions?

The manuscript must describe a technically sound piece of scientific research with data that supports the conclusions. Experiments must have been conducted rigorously, with appropriate controls, replication, and sample sizes. The conclusions must be drawn appropriately based on the data presented.

Reviewer #1: (No Response)

Reviewer #2: (No Response)

**********

3. Has the statistical analysis been performed appropriately and rigorously?

Reviewer #1: (No Response)

Reviewer #2: (No Response)

**********

4. Have the authors made all data underlying the findings in their manuscript fully available?

The PLOS Data policy requires authors to make all data underlying the findings described in their manuscript fully available without restriction, with rare exception (please refer to the Data Availability Statement in the manuscript PDF file). The data should be provided as part of the manuscript or its supporting information, or deposited to a public repository. For example, in addition to summary statistics, the data points behind means, medians and variance measures should be available. If there are restrictions on publicly sharing data—e.g. participant privacy or use of data from a third party—those must be specified.

Reviewer #1: (No Response)

Reviewer #2: (No Response)

**********

5. Is the manuscript presented in an intelligible fashion and written in standard English?

PLOS ONE does not copyedit accepted manuscripts, so the language in submitted articles must be clear, correct, and unambiguous. Any typographical or grammatical errors should be corrected at revision, so please note any specific errors here.

Reviewer #1: (No Response)

Reviewer #2: (No Response)

**********

6. Review Comments to the Author

Please use the space provided to explain your answers to the questions above. You may also include additional comments for the author, including concerns about dual publication, research ethics, or publication ethics. (Please upload your review as an attachment if it exceeds 20,000 characters)

Reviewer #1: Congratulations for the very high quality and detailed revision. All sections and aspects of the paper are improved, and all of my comments and concerns are addressed. The paper is much stronger, and it is ready for publication.

Reviewer #2: (No Response)

**********

7. PLOS authors have the option to publish the peer review history of their article (what does this mean?). If published, this will include your full peer review and any attached files.

If you choose “no”, your identity will remain anonymous but your review may still be made public.

**Do you want your identity to be public for this peer review?**    For information about this choice, including consent withdrawal, please see our Privacy Policy.

Reviewer #1: No

Reviewer #2: **Yes:**    Lauren Parlett

**********

# Editor report: Acceptance letter

**DOI:** 10.1371/journal.pone.0351075.r004

**Authors:** Sina Azadnajafabad

PONE-D-25-65931R1

PLOS One

Dear Dr. Cromer,

I'm pleased to inform you that your manuscript has been deemed suitable for publication in PLOS One. Congratulations! Your manuscript is now being handed over to our production team.

At this stage, our production department will prepare your paper for publication. This includes ensuring the following:

* All references, tables, and figures are properly cited

* All relevant supporting information is included in the manuscript submission,

* There are no issues that prevent the paper from being properly typeset

You will receive further instructions from the production team, including instructions on how to review your proof when it is ready. Please keep in mind that we are working through a large volume of accepted articles, so please give us a few days to review your paper and let you know the next and final steps.

Lastly, if your institution or institutions have a press office, please let them know about your upcoming paper now to help maximize its impact. If they'll be preparing press materials, please inform our press team within the next 48 hours. Your manuscript will remain under strict press embargo until 2 pm Eastern Time on the date of publication. For more information, please contact onepress@plos.org.

You will receive an invoice from PLOS for your publication fee after your manuscript has reached the completed accept phase. If you receive an email requesting payment before acceptance or for any other service, this may be a phishing scheme. Learn how to identify phishing emails and protect your accounts at https://explore.plos.org/phishing.

If we can help with anything else, please email us at customercare@plos.org.

Thank you for submitting your work to PLOS ONE and supporting open access.

Kind regards,

PLOS ONE Editorial Office Staff

on behalf of

Dr. Sina Azadnajafabad

Academic Editor

PLOS One
